# Nitric Oxide-Mediated Maize Root Apex Responses to Nitrate are Regulated by Auxin and Strigolactones

**DOI:** 10.3389/fpls.2015.01269

**Published:** 2016-01-22

**Authors:** Alessandro Manoli, Sara Trevisan, Boris Voigt, Ken Yokawa, František Baluška, Silvia Quaggiotti

**Affiliations:** ^1^Department of Agronomy, Food, Natural Resources, Animals and Environment, University of PaduaPadua, Italy; ^2^Department of Plant Cell Biology, Institute of Cellular and Molecular Botany, University of BonnBonn, Germany; ^3^Department of Biological Sciences, Tokyo Metropolitan UniversityTokyo, Japan

**Keywords:** root, transition zone, nitrate, nitric oxide, auxin, strigolactones

## Abstract

Nitrate (NO_3_^-^) is a key element for crop production but its levels in agricultural soils are limited. Plants have developed mechanisms to cope with these NO_3_^-^ fluctuations based on sensing nitrate at the root apex. Particularly, the transition zone (TZ) of root apex has been suggested as a signaling-response zone. This study dissects cellular and molecular mechanisms underlying NO_3_^-^ resupply effects on primary root (PR) growth in maize, confirming nitric oxide (NO) as a putative modulator. Nitrate restoration induced PR elongation within the first 2 h, corresponding to a stimulation of cell elongation at the basal border of the TZ. Xyloglucans (XGs) immunolocalization together with Brefeldin A applications demonstrated that nitrate resupply induces XG accumulation. This effect was blocked by cPTIO (NO scavenger). Transcriptional analysis of *ZmXET1* confirmed the stimulatory effect of nitrate on XGs accumulation in cells of the TZ. Immunolocalization analyses revealed a positive effect of nitrate resupply on auxin and PIN1 accumulation, but a transcriptional regulation of auxin biosynthesis/transport/signaling genes was excluded. Short-term nitrate treatment repressed the transcription of genes involved in strigolactones (SLs) biosynthesis and transport, mainly in the TZ. Enhancement of *carotenoid cleavage dioxygenases* (*CCD*s) transcription in presence of cPTIO indicated endogenous NO as a negative modulator of CCDs activity. Finally, treatment with the SLs-biosynthesis inhibitor (TIS108) restored the root growth in the nitrate-starved seedlings. Present report suggests that the NO-mediated root apex responses to nitrate are accomplished in cells of the TZ via integrative actions of auxin, NO and SLs.

## Introduction

Nitrogen (N) is one of the most important elements for plant life. In soil, N is present in different N-containing compounds. Under aerobic soil conditions, soluble nitrate (NO_3_^-^) is the major N source taken up by crop roots (Krouk et al., 2010; Wang et al., 2012). In well-aerated agricultural soils, NO_3_^-^ concentrations are extremely variable in time and space (Miller et al., 2007) and current agricultural practices strongly depend on massive applications of synthetized N fertilizers (Erisman et al., 2008; Robertson and Vitousek, 2009). Due to the low Nitrogen Use Efficiency (NUE) of crops (Raun and Johnson, 1999; Baligar et al., 2001; Robertson and Vitousek, 2009), more than 50% of the available N is lost from the plant-soil system. This leads to serious concerns about biosphere pollution and human health (Erisman et al., 2013; Fowler et al., 2013; Galloway et al., 2013, and references therein). Therefore the understanding of the molecular and physiological events underlying plant adaptation to NO_3_^-^ fluctuations is crucial in order to improve plant NUE and to reduce negative impacts on environment and humans.

Besides being an essential nutrient, NO_3_^-^ acts as a signal regulating metabolism and development in plants (López-Bucio et al., 2003; Vidal and Gutiérrez, 2008; Ho et al., 2009; Prinsi et al., 2009; Krouk et al., 2010; Bouguyon et al., 2012; Wang et al., 2012; Vidal et al., 2013; Ruiz Herrera et al., 2015). Considering that the root system architecture (RSA) determines the plasticity of plants to explore the soil for searching water and nutrients, investigation on the RSA and root morphology are very important. Despite a huge number of reports on NO_3_^-^ effects on RSA in model plants, crop’s studies are still fragmentary (Hirel et al., 2007).

Numerous papers report connections between auxin (IAA) and NO_3_^-^ in the control of primary root (PR) growth and both lateral root (LR) development (reviewed in López-Arredondo et al., 2013). Long distance transport of IAA from shoot to root was proposed to be involved in the inhibition of early LR development by high rates of NO_3_^-^ in *Arabidopsis* (Forde, 2002; Walch-Liu et al., 2006). A role of IAA was also evidenced in maize, in which high NO_3_^-^ supply inhibited root growth by lowering the IAA levels in roots (Tian et al., 2008). More recently, the involvement of IAA in the LR growth regulation by NO_3_^-^ was reconsidered when the *Arabidopsis* NO_3_^-^ transporter NRT1.1 was demonstrated to be able to move IAA as well as NO_3_^-^ (Krouk et al., 2010). Furthermore, NO_3_^-^ was recently proposed to regulate downstream root architecture adjustments in maize through the fine-tuning control of nitric oxide (NO) production and scavenging dependent on the coordinated activities of nitrate reductase (NR) and non-symbiotic hemoglobins (nsHbs) (Trevisan et al., 2011, 2014; Manoli et al., 2014), which takes preferentially place in the transition zone (TZ) of root apex. TZ cells are highly sensitive to touch and extracellular calcium (Ishikawa and Evans, 1992; Baluška et al., 1996), aluminum (Marciano et al., 2010; Sivaguru et al., 2013; Yang et al., 2014), osmotic stress (Baluška and Mancuso, 2013), auxin (Mugnai et al., 2014) and gravity (Masi et al., 2015). This high TZ sensitivity to environmental signals make it a sort of information processing and control center, allowing the growing root apex to monitor the rhizosphere in real time and to elicit proper responses (Baluška et al., 2010; Baluška and Mancuso, 2013).

The involvement of NO in root development was postulated also in *Arabidopsis* (reviewed by Sanz et al., 2015), being at least in part associated with auxin actions (Correa-Aragunde et al., 2004). Fernández-Marcos et al. (2011) reported inhibitory effects of NO on rootward polar transport, due to a PIN1 depletion and decreased numbers of dividing cells in the PR meristem. They also demonstrated that during early root development endogenous NO accumulates mainly in a zone situated between the apical meristem and the elongation zone, namely the TZ. Recently Sanz et al. (2014) demonstrated that the auxin biosynthesis, transport, and signaling are perturbed in *noa1* and *nia1 nia2 noa1* NO-deficient mutant roots.

Other studies demonstrated that endogenous NO application affects PR growth by reducing the pool of dividing cells in the root apical meristem, causing a reduction in cell-division rates and an increase in cell lengths of the meristem (Méndez-Bravo et al., 2010; Fernández-Marcos et al., 2011). These results suggest that high levels of NO caused by environmental stimuli or elicitors could positively regulate the exit of cells from the PR meristem and the TZ into the elongation and differentiation zones, altering the PR growth.

Moreover NO was inferred to act as a master regulator of primary maize root growth by affecting the functioning of the actin cytoskeleton and actin-dependent mechanisms (Kasprowicz et al., 2009) and modulating cell wall biosynthesis contributing to modification of cell growth (Yu et al., 2014). The present study was aimed to gain new knowledge on the cellular and molecular mechanisms underlying the NO-mediated nitrate action on *Zea mays* L. PR growth. Confocal microscopy was applied, along with morphometric analysis, in order to evaluate the effect of nitrate supply on PR growth and any cell size modification. IAA, PIN1 and xyloglucan (XG) distributions were examined after immunostaining, and transcriptomic analyses under nitrate applications were also carried on. The results indicate that PR growth stimulation by short term nitrate provision could be attributed to a putative interference with the basipetal (shootward) auxin flow and the XG deposition, particularly affecting cell expansion in the TZ. Furthermore, strigolactones (SLs) and NO seem to act upstream of this signaling, via regulating the balance between cell division and expansion.

## Materials and Methods

### Maize Growth Conditions

Seeds of the maize inbred line B73 were germinated in paper rolls soaked with distilled water and then transferred to hydroponic systems as described in Manoli et al. (2014). Seedlings were grown for 24 h in a nitrate depleted solution (Manoli et al., 2014) and then transferred in either a nitrate supplied (+N, 1 mM) or a nitrate depleted solution (-N). Root growth measurements were carried out at 2, 6, 24, and 48 h after the transfer. For gene expression analysis, immunolocalization and *in situ* hybridization, tissues were collected after 2 h and immediately frozen (-80°C).

Treatments with 1 mM 2-(4-carboxyphenyl)- 4,4,5,5-tetramethylimidazoline-1-oxyl-3-oxide (cPTIO) in combination with NO_3_^-^ and with 0.01 mM sodium nitroprusside (SNP) (Manoli et al., 2014) were also set up to better evaluate the NO role. For the Brefeldin A (BFA) treatment, a solution diluted in a phosphate-buffered saline (PBS) to achieve an effective working solution of 100 μM was utilized.

6-phenoxy-1-phenyl-2-(1H-1,2,4-triazol-1-yl) hexan-1-one (TIS108) (Strigolab, Torino, Italy) was used at a 2 μM concentration as inhibitor of SL biosynthesis (Ito et al., 2011). Unless stated otherwise, all chemicals were obtained from Sigma Chemicals (Sigma, St Louis, MO, USA). The zonation correspond to: meristem (0.5–2 mm from the root cap tip), the TZ (2–4 mm from the root cap tip), the rapid elongation zone (4–8 mm from the root cap tip) and the maturation zone (the residual portion).

### RNA Extraction and cDNA Synthesis

Primary root portions were harvested from 15 to 20 pooled seedlings, in three independent biological repetitions. Tissues were ground in liquid nitrogen and RNA was extracted using TRIzol reagent (Invitrogen, San Giuliano Milanese, Italy) as previously described by Trevisan et al. (2011). An aliquot of total RNA was treated with RQ1 RNAse-free DNAse (Promega, Milano, Italy) as described by Trevisan et al. (2011). RNA was quantified with a Nanodrop1000 (Thermo Scientific, Nanodrop Products, Wilmington, DE, USA) and cDNA was synthesized from 500 ng of total RNA mixed with 1 μl of 10 μM oligo-dT, as described by Manoli et al. (2012).

### Real-Time Quantitative PCR

To confirm gene expression levels detected by RNA-Seq and to further investigate gene expression in other root portions, qRT-PCR was performed using the StepOne Real-Time PCR System (Applied Biosystems, Monza, Italy) as described by Nonis et al. (2008), using SYBR Green reagent (Applied Biosystems, Monza, Italy), according to the manufacturer’s instructions. Target gene relative expression was determined according to the Livak and Schmittgen (2001) method, using *LUG* (leunig primers, forward 5′-TCCAGTGCTACAGGGAAGGT-3′ and reverse 5′-GTTAGTTCTTGAGCCCACGC-3′) and *MEP* (membrane protein PB1A10.07c, primers: forward 5′-TGTACTCGGCAATG CTCTTG-3′ and reverse 5′-TTTGATGCTCCAGGCTTACC-3′), as reference genes, according to Manoli et al. (2012). Primers used in qRT-PCR were designed using Primer3 web tool (version 0.4.0; http://frodo.wi.mit.edu/primer3/; Rozen and Skaletsky, 2000) and further verified with the PRATO web tool (Nonis et al., 2011) and are listed in **Supplementary Table S1**. See Forestan and Varotto, 2010; Forestan et al., 2012 for primers used in RT-PCR *PINs* amplification). Three technical replicates were performed on three independent biological repetitions.

### RNA *In Situ* Hybridization

*In situ* hybridization of maize PR with digoxigenin-labeled probes was performed as described by Trevisan et al. (2011). GRMZM2G145008 was amplified as probe in PCR using the primers listed in **Supplementary Table S1**. The fragment was cloned into the T-easy vector (Madison, WI, USA) for labeling. The sense and antisense probes were synthesized *in vitro* using T7 and SP6 RNA polymerases (Roche, Basel, Switzerland) and labeled with digoxygenin (DIG) RNA labeling mix (Roche, Basel, Switzerland) following the manufacturer’s protocol. Root tissues were fixed overnight in RNase-free 4% formaldehyde. Samples were dehydrated in a graded ethanol series, embedded in Paraplast Plus (Sigma, St Louis, MO, USA) and sectioned (7 μm) as described previously (Trevisan et al., 2011). After hybridization and staining, slides were observed with an Olympus BX50 microscope (Olympus Corporation, Tokyo, Japan). Images were captured with an Axiocam Zeiss MRc5 color camera (Carl Zeiss, Oberkochen, Germany), and processed with Adobe Photoshop 6.0.

### Primary Root Length Analysis, Cell Length Measurement and Confocal Microscopy

To monitor the influence of different nitrate availabilities on root length, maize seedlings grown for 24 h in NO_3_^-^ depleted solution were scanned (T0) and then transferred to either a nitrate supplied or depleted solution. The root length of each seedlings was monitored at several time points (2, 6, 24, 48 h). Images were taken at each determined time points of the experimental plan using a flatbed scanner. This allowed the root systems of 10 seedlings to be imaged simultaneously. The PR length was measured using the Image J Image Analysis Software and the increment in root length was calculated. Three biological replicates were performed for each treatment (*n* = 30).

To measure cortex cell length and density, whole roots were first stained with propidium iodide (Sigma, St Louis, MO, USA) and then washed in sterile water twice. Pictures were taken with a Zeiss LSM 510 confocal microscope. Cortex cells were numbered in a region of 100 μm^2^, and their length was averaged from at least 10 cells per snapshot at a distance of 0.5 mm from the root tips from at least ten roots examined for each treatment. Cell length was measured using Zeiss LSM 510 software.

### Indirect Immunofluorescence Labeling

Apical root segments (8–10 mm) were excised into 3.7% formaldehyde prepared in stabilizing buffer (MTSB) (50 mM piperazine-N,N′-bis (2-ethanesulfonic acid), 5 mM MgSO_4_, 5mM EGTA; pH 6.9) and vacuum infiltrated for 1 h at room temperature (RT). After an overnight rinse in MTSB at 4°C, the root apices were dehydrated in a graded ethanol series diluted with PBS (pH 7.3), embedded in Steedman’s wax and processed for immunofluorescence (for details, see Baluška et al., 1990).

Sections were then incubated with the following primary antibodies (diluted in PBS and supplemented with 1% BSA): anti-XG antibodies diluted 1:200 (Sonobe et al., 2000), anti-IAA monoclonal antibodies diluted 1:20 and anti-PIN1 polyclonal antibodies diluted 1:40 for 1 h at RT. After rinsing in PBS, the sections were incubated for 1 h with FITC-conjugated anti-rabbit IgGs (Sigma, St Louis, MO, USA), each raised in goat and diluted 1:100 in buffer containing 1% BSA. A further wash with PBS (10 min) preceded both a 10 min staining with 4**′**,6-diamino-2-phenylindole dihydrochloride (DAPI; 100 μM in PBS) and a 10 min treatment with 0.01% Toluidine Blue. The sections were then mounted using an anti-fade mounting medium containing *p*-phenylenediamine (Baluška et al., 1990) and examined with an Axiovert 405M inverted microscope (Zeiss, Oberkochen, Germany) equipped with epifluorescence and standard fluorescein isothiocyanate excitation and barrier filters.

## Results

### Primary Root Elongation is Stimulated by Short-Term Nitrate Supply

**FIGURE 1:**
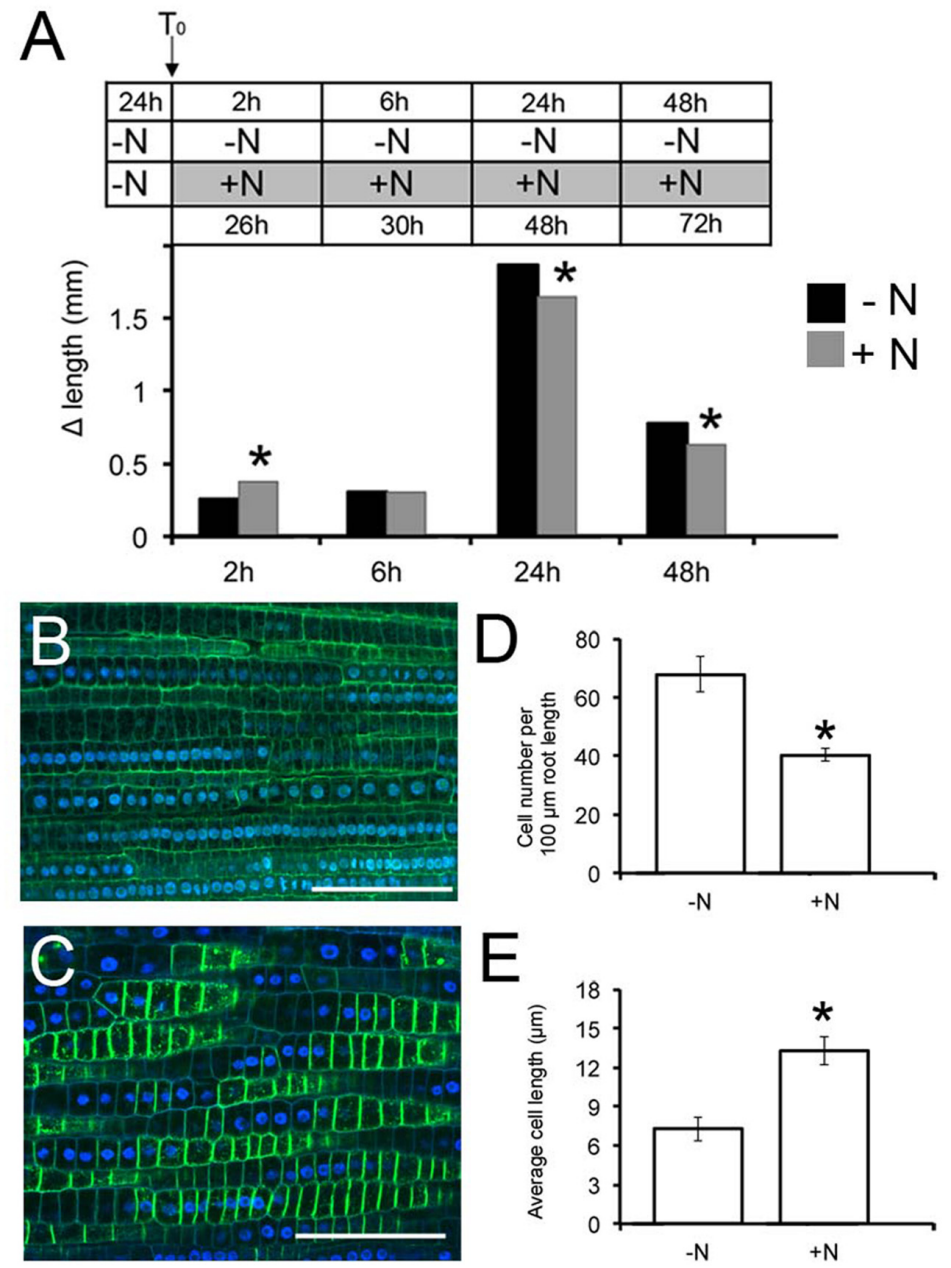
**Nitrate availability modulates *Zea mays* L. primary root (PR) growth.** Two days maize seedlings were grown for 24 h in -NO_3_^-^ solution and then transferred to + NO_3_^-^**(A,C)** or – NO_3_^-^**(A,B)** medium (T_0_). PR length was measured at T_0_ and after 2, 6, 24, and 48 h of nitrate depletion/provision. The time course of Δ length respect to the T_0_ was calculated **(A)**. Data are means ± SE of three biological replicates. Confocal images (derived from XG labeling) were analyzed to determine the cortical cell number in the transition zone (TZ) of each plant after 2 h of NO_3_^-^ supply **(C)** depletion **(B)**. **(B,C)** were derived from XG labeling pictures. Histograms summarizing quantification of cortical cell number **(D)** and calculation of the average cortex cell length **(E)** in the cortex of maize root TZ. Results are presented as mean ± SE from three experiments (*n* = 5–10). Bar = 100 μm. Asterisk indicates significant differences, *P* < 0.01, based on ANOVA.

The effects of nitrate supply on PR growth were tested on maize seedlings grown for 1 day in a nitrate depleted solution (T_0_), before either being transferred into a similar solution, or to a nitrate supplied solution. The root length was monitored at different time points (2, 6, 24 and 48 h) and the increments (Δ length) were then calculated (**Figure 1A**).

Root growth responded to nitrate provision, generating significantly elongated PRs compared to seedlings grown on the nitrate depleted solution (**Figure 1A**), already after 2 h of nitrate provision. The protracted exposure (6 h) to nitrate induced a gradual decline of PR growth respect to the negative control, with a significant inhibition after longer periods of starvation (24 and 48 h). The 2 h treatment was chosen to further characterize the earlier effects of nitrate on root growth.

### Nitrate-Mediated Increase in Primary Root Length Reflects Increase in Transition Zone Size

Cell division and elongation contributes to PR growth. To interpret the increased growth exhibited early after nitrate provision, the number and the length of cortex cells were determined in the root TZ (the region between meristem and elongation zone) by staining with propidium iodide and observation under a confocal microscope. Application of 1 mM nitrate resulted in a significant (40%) decrease in the number of cells in this region compared to the negative control (**Figures 1B–D**). In contrast, the length of cortical cells was increased by 2 fold in NO_3_^-^-fed roots compared with seedlings grown on N-free medium (**Figures 1B,C,E**), indicating that nitrate availability controls the onset of rapid cell elongation, rather than cell division, giving rise to an increased size of the TZ.

To determine whether the increase of PR growth after nitrate treatment might be related to differences in root meristem length, the size and number of cells were determined in the meristem root zone (**Supplementary Figure S1**). We found that both the number and the length of cells in the meristem region were not significantly affected by nitrate provision, straightening the hypothesis that the nitrate-mediated increase in PR length is dependent on an increase in TZ size.

### Xyloglucans Modification and ZmXET1 Expression are Induced Concomitantly in Response to Nitrate Provision

**FIGURE 2:**
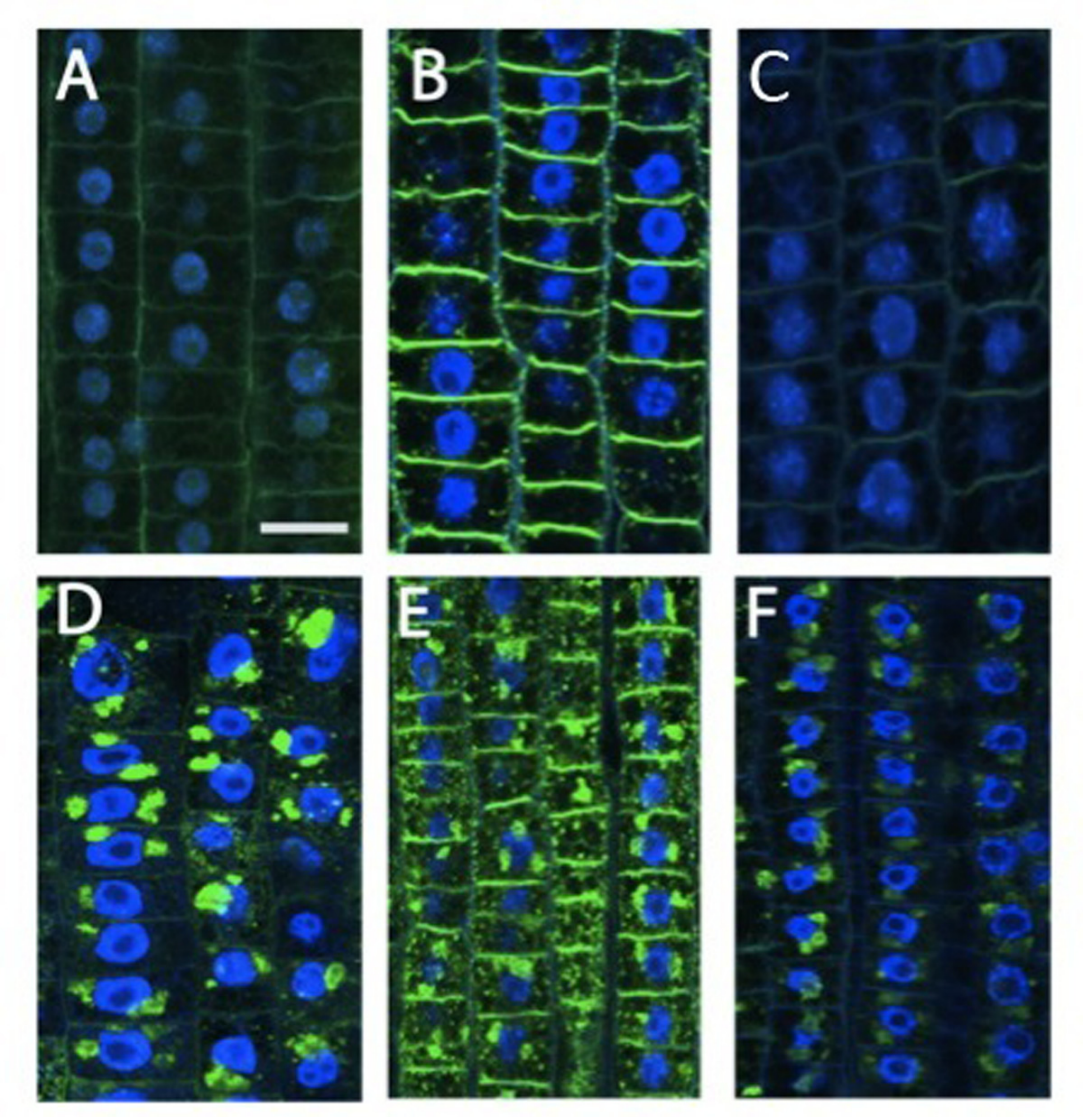
**Immunolocalization of xyloglucans (green staining) in cells of root **TZ**.** The blue-emitting fluorescence is due to DAPI (4′,6-diamidino-2-phenylindole) compound which was used to better characterized nuclear staining. **(B)** Nitrate treatment resulted in a very abundant accumulation of XGs, especially in cross walls, in comparison with the negative control **(A)** and + NO_3_^-^ roots treated with cPTIO **(C)**. **(D–F)** In BFA-treated cells, almost all XGs internalized into BFA compartments. **(D)** Roots grown in a nitrate-depleted solution, or **(E)** nitrate-resupply solution, or **(F)** nitrate-resupply and cPTIO solution. Bars: for **(A,B,D)** 18 μm; for **(F)** 20 μm; for **(E)** 22 μm.

Beside cytoskeletal organization, several genes related to cell wall structure and composition, including those encoding XGs, endotransglycosylases, polygalacturonases, and glycosyltransferases, have been previously identified as being involved in the regulation of early nitrate response in root TZ (Trevisan et al., 2015). Moreover, root TZ also shows the highest rate of endocytic vesicle recycling activity in recycling XGs from cell walls into BFA-induced compartments, as demonstrated by Baluška et al. (2005). To further investigate the cell wall rearrangements induced by nitrate supply, the distribution of XGs were analyzed. XGs visualized by labeling with antibodies were abundant, especially at the cell walls that resulted as the main site of XG accumulation. This pattern of tissue-dependent immunofluorescence was observed both in nitrate-depleted (**Figure 2A**) and nitrate-supplied maize roots (**Figure 2B**); however, immunofluorescence signal was generally significantly stronger in the root subjected to nitrate treatment (**Figure 2B**), and this was more evident at the cross-wall (end-poles) domains. Moreover, to test XGs recycling within cells, BFA was supplied for 2 h to both nitrate-supplied (**Figure 2E**) and nitrate-depleted roots (**Figure 2D**). In -N roots, almost all XGs were removed from cell walls into BFA-compartments and, in particular, cross walls (end-poles) of root TZ cells showed very weak signal after BFA-treatment, in comparison to TZ cells of nitrate-supplied roots (**Figure 2D**). Indeed in the latter case, BFA-treatment partially failed in removing all XGs from cell walls and a marked signal was still visible, especially at the cross wall domains. To better understand the role of NO in nitrate signaling the effect of a NO scavenger (cPTIO) was evaluated. Nitrate-fed roots supplied with cPTIO (**Figure 2C**) showed a behavior similar to that observed for nitrate-depleted cells (**Figure 2A**).

**FIGURE 3:**
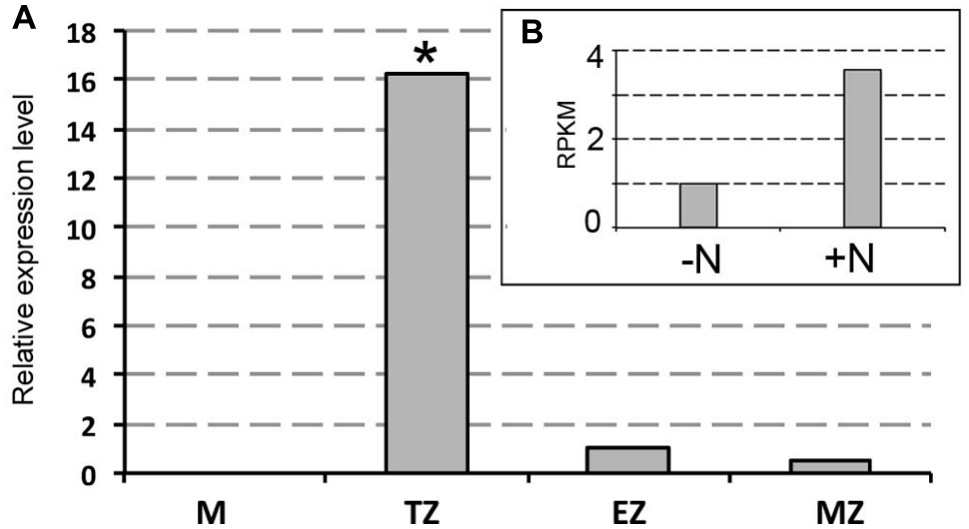
**Real-time quantitative PCR analysis of mRNA encoding ZmXET1 (GRMZM2G026980).** **(A)** Real time validation and investigation of *ZmXET1* in the four root portions (M, meristem; TZ, transition zone; EZ, elongation zone; MZ, mature zone). Data are expressed as ratio of +N/-N relative expression values. Quantitative real-time PCR was performed in triplicate (three biological replicates). Asterisk indicates significant differences between the treatments, *P* < 0.01, based on ANOVA. **(B)** RNA-Seq RPKM values in maize root TZ of 2 days seedlings grown for 24 h in a NO_3_^-^ depleted solution and then moved to a NO_3_^-^ supplied (+N) or deprived (-N) solution for 2 h.

Previous works have shown as XG endotransglycosylase, a key enzyme in cleaving XGs chains, had the most prominent activity in the TZ in both maize and *Arabidopsis* roots (Pritchard et al., 1993 and Vissenberg et al., 2000, respectively). Transcriptional analysis of the XG endotransglucosylase *ZmXET1* (*GRMZM2G026980*) was performed by quantitative real-time PCR method to monitor differences between expression levels after 2 h of nitrate supply or deprivation in the four different root portions (**Figure 3**). The expression profile of *ZmXET1*, encoding the XG endotransglucosylase (GRMZM2G026980) previously identified (Trevisan et al., 2015) evidenced an up-regulation upon short-term nitrate provision (**Figure 3B**). Real Time analysis here carried out confirmed this result and showed an induction of more than 10 times of its expression after 2 h of nitrate treatment in cells of TZ (**Figure 3A**). On the contrary, not significant differences of expression were measured in the other three root portions.

### Auxin-Related Gene Expression is Not Significantly Changed Under Short-Term Nitrate Starvation

**FIGURE 4:**
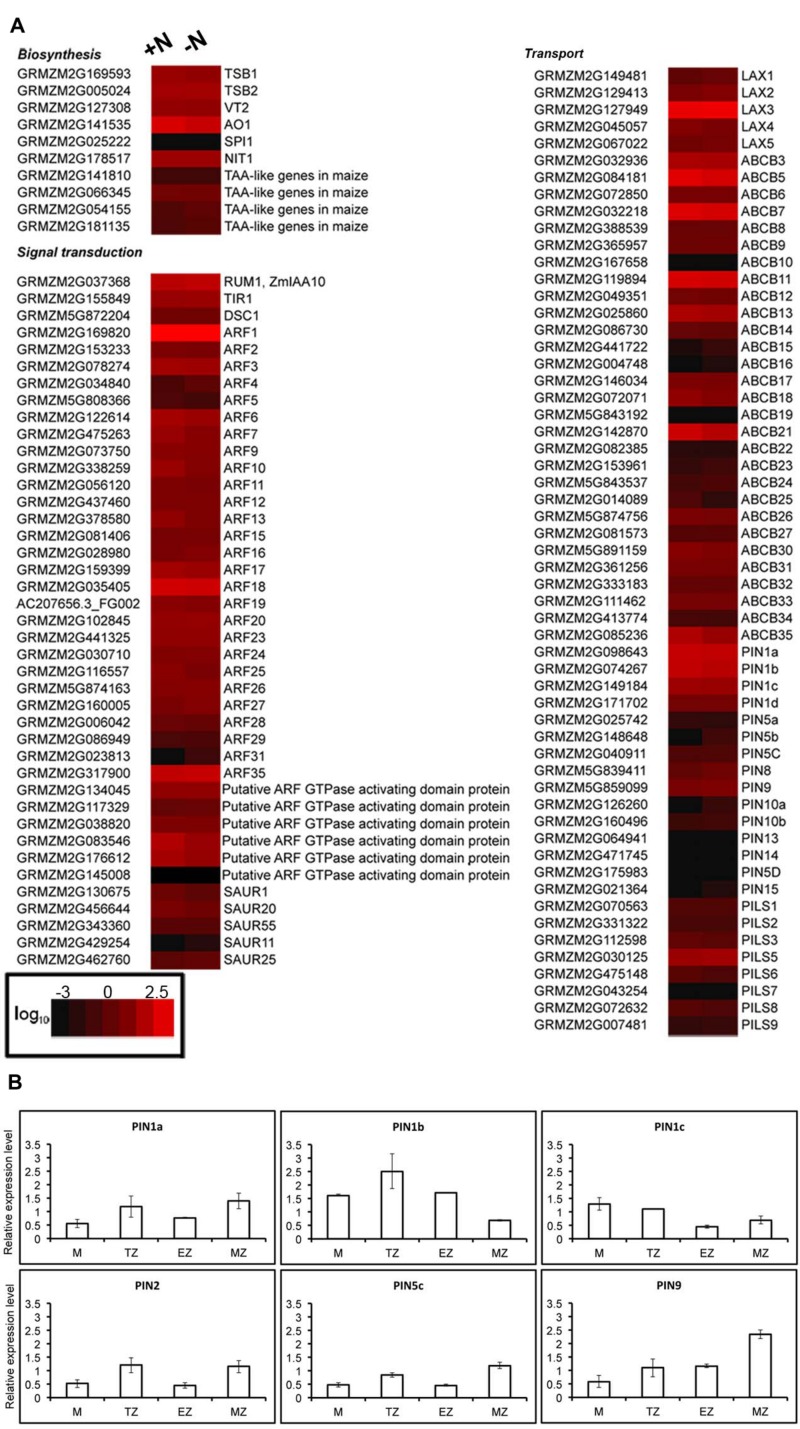
**Nitrate does not affect accumulation of auxin related transcripts in root TZ.** **(A)** RNA-Seq analysis of transcript levels of transcripts involved in auxin biosynthesis, signaling and transport in the roots of 2-day-old plants grown in a nitrate-depleted solution for 24 h and treated without (-N) or with (+N) 1 mM NO_3_^-^ for 2 h. Colors indicate the range of each gene expression, with least expression shown in black and highest expression shown in red. Transcripts abundance represented by the heat map are the average of transcript abundance values from three independent. Data shown are expressed as log_10_ of RPKM values of the RNA-seq analysis (Trevisan et al., 2015) and are means of two independent experiments. **(B)** Quantitative RT-PCR validation of RNA-seq expression profiling of six *PIN*s transcripts (*ZmPIN1a, ZmPIN1b, ZmPIN1c, ZmPIN2, ZmPIN5c, ZmPIN9)* in various root portions. The levels of *PIN*s transcripts were measured in total RNAs from: meristem-enriched zone (<3 mm from the root tip); TZ-enriched portion (the next 0.8 cm); elongation zone-enriched portion (the next 0.8 cm); and maturation zone (the residual portion). Expression levels are expressed as +N/-N fold change in the four zones of nitrate-supplied seedlings (+N) relative to nitrate deprived seedlings (-N).

Both localization and size of the TZ are largely controlled by the local establishment of an auxin gradient that regulates the balance between cell proliferation and cell elongation (Blilou et al., 2005). Meta-analysis of a published RNA-seq data that investigated the transcriptome changes in response to 2 h of nitrate provision after 24 h of nitrate starvation in maize root TZ (Trevisan et al., 2015) has been used to investigate the auxin-mediated nature of nitrate action on PR growth. To focus on relevant auxin-related responses, the expression values of a set of genes involved in auxin biosynthesis, signaling and transport were searched. The results showed that in the TZ the IAA-related genes expression is not significantly altered in response to nitrate (**Figure 4A**). Furthermore, the expression of a set of *Zea mays* auxin efflux carriers PIN family members (PIN1a, PIN1b, PIN1c, PIN2, PIN5c, and PIN9) was measured in all the four root portions, but only slight differences were evidenced between nitrate supplied and nitrate deprived plants (**Figure 4B**).

### Nitrate Provision Induces Auxin Accumulation at Cross Wall in Root Apex Transition Zone

**FIGURE 5:**
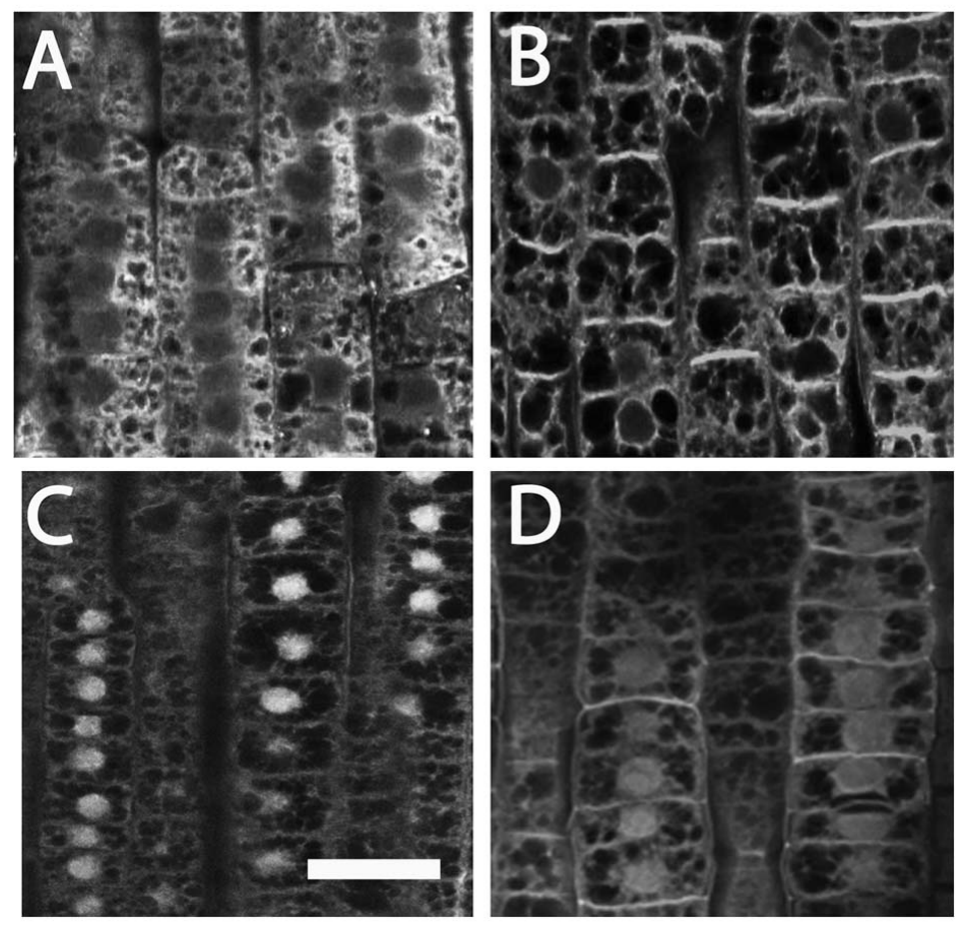
**Immunolocalization of auxin **(A,B)** and PIN1 **(C,D)** in cells of maize root TZ.** **(A)** The localization of auxin (IAA) in -NO_3_^-^ roots showed that a prominent IAA signal was visible at the cytoplasm. **(B)** Exposure of root apices to NO_3_^-^ resulted in a strong immunofluorescence at the cross walls (end-poles). **(C)** A prominent PIN1 signal was scored within nuclei in -NO_3_^-^ root cells. **(D)** In the NO_3_^-^ treated roots, PIN1 labeling within nuclei slightly vanished while end-poles was clearly enriched with PIN1. Bar in **(C)**: for **(A,C)** 20 μm; for **(D)** 18 μm; for **(B)** 16 μm.

To better investigate the putative involvement of auxin in nitrate action on maize root apex, auxin immunolocalization were performed on roots nitrate supplied or deprived. Nitrate deprived roots showed a prevalent IAA accumulation in the cytoplasm, while weaker signal was detected around nuclei (**Figure 5A**). A slight increase of signal around nuclei and a strong immunofluorescence at the cross walls (end-poles) were, instead, observed after exposure of root apices to 1 mM nitrate for 2 h (**Figure 5B**).

Immunostaining experiments using an anti-PIN1 antibody evidenced a prominent PIN1 signal within nuclei in TZ cells of nitrate depleted roots (**Figure 5C**), which slightly vanished after nitrate provision (5D). On the contrary, almost all end-poles were strongly enriched with PIN1 in nitrate treated roots (**Figure 5D**), co-localized with the auxin immunofluorescence signal (**Figure 5B**).

### Nitrate Provision Affects Transcription of Genes Involved in Cytoskeletal Organization

Eleven transcripts putatively involved in cytoskeletal organization showed a significant regulation by short-term nitrate provision (Trevisan et al., 2015; **Table 1**). A large part of them (8) were up-regulated by 2 h of nitrate supply. The gene ontology classification of their biological process grouped them as cytoskeletal protein binding, microtubule cytoskeleton organization, tubulin binding, vesicle-mediated transport, ARF GTPase activator activity (**Table 1**). According to its putative relevance, *GRMZM2G145008* was selected among all the DEGs analyzed to be further studied and the spatial expression patterns of its transcript was precisely mapped by means of *in situ* hybridizations on longitudinal sections of PR apices of maize seedlings. The transcript *GRMZM2G145008* encodes an unknown protein containing a GTPase activating protein domain for Arf (ARFGAP domain), with predicted orthology with the *Arabidopsis* ARF GAP-like zinc finger-containing protein *AGD14* gene.

**Table 1:** RNA seq expression profile of differentially expressed genes (DEGs) significantly responsive to 1 mM NO_3_^-^ exposure (*P*-values ≤0.01) concerned with cytoskeletal organization (Trevisan et al., 2015).

Accession	-N	+N	Description	Gene ontology annotation (BP)
GRMZM2G140455	60.2	24	Profilin	Cytoskeletal protein binding
GRMZM5G860469	4.7	1.3	ATP binding	Microtubule motor activity
GRMZM2G112782	29.3	10.5	Uncharacterized protein	Vesicle-mediated transport
GRMZM2G318849	0.8	2.3	Uncharacterized protein	Microtubule-based process
GRMZM2G082484	46	19	Putative actin	ATP binding
GRMZM2G045808	0.1	0.8	Uncharacterized protein, Microtubule-associated protein RP/EB protein domain	Cytoskeletal protein binding
GRMZM2G166082	1.1	2.8	Uncharacterized protein	Small GTPase mediated signal transduction
GRMZM2G015886	1.6	4.1	Putative cellulose synthase-like	Microtubule cytoskeleton organization
GRMZM2G145008	0.02	0.4	Uncharacterized protein	ARF GTPase activator activity
GRMZM2G147942	6.3	14	Subtilisin-like serine protease 2	Cytoskeleton organization
GRMZM2G045808	0.1	0.8	Microtubule-associated protein RP/EB family member 3	Tubulin binding


*Differentially expressed genes are reported together with their gene ontology classification.*

**FIGURE 6:**
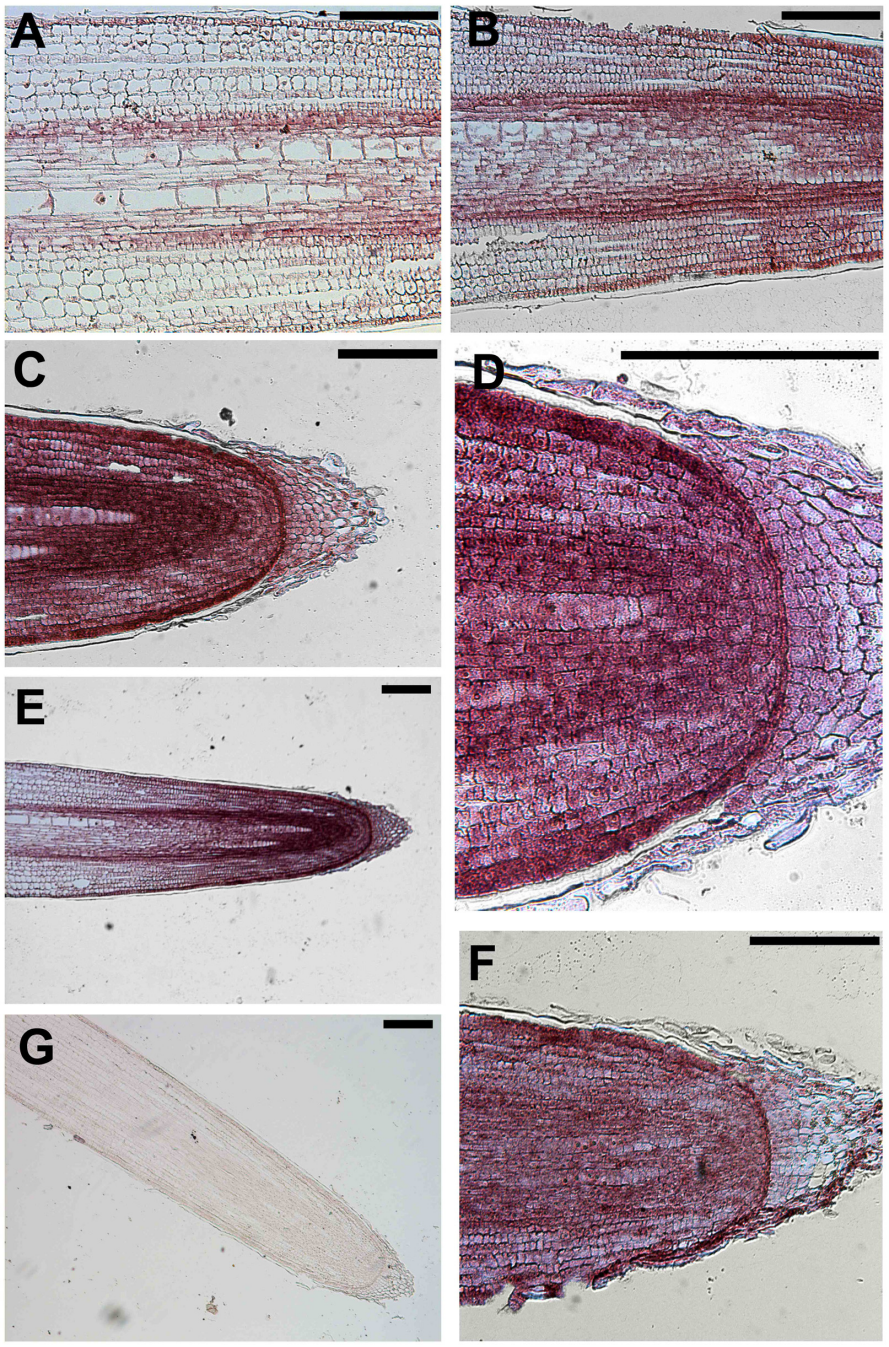
***In situ* mRNA hybridization of maize root with DIG-labeled antisense probes of *GRMZM2G145008*.** All images represent longitudinal sections of B73 inbred line root apexes of 2 days maize seedlings grown for 24 h in a NO_3_^-^ depleted solution and then moved to a NO_3_^-^ supplied solution. The hybridization signal is represented by the red-purple staining. The figure shows sections hybridized with antisense probes **(A–F)** and with sense probes **(G)** (negative controls). Longitudinal sections through a primary maize root hybridized with *GRMZM2G145008* antisense transcript showing the hybridization signal in root TZ **(A,B,E)**, meristem **(C–F)**, elongation zone **(E)**. **(C)** Portrays a magnification of the root cap and **(D)** represents a higher magnification of **(C)** showing the purple staining in the cells of the root meristem. Bars, 200 μm.

The sense probe served as negative control and did not generate a detectable hybridization signal (**Figure 6G**). In contrast, the purple/blue signal arising from the antisense probe revealed the strongest expression of this gene in epidermal cells of the apical root zone, mainly expressed in the apical meristem and in the TZ, above the root cap (**Figures 6C–F**). Moreover, the transcript signal was also abundant in the stele of roots and particularly in the pericycle and endodermis layers along the central cylinder/cortex boundary of the meristem and the TZ (**Figures 6B,E**). Regarding its longitudinal distribution, expression was highest in the root tips, but, interestingly, a signal was also detected in epidermal cells of the elongation zone (**Figure 6A**).

### Nitrate Provision Represses Transcription of Genes Involved in SL Biosynthesis and Transport in Cells of Root Apex Transition Zone

**FIGURE 7:**
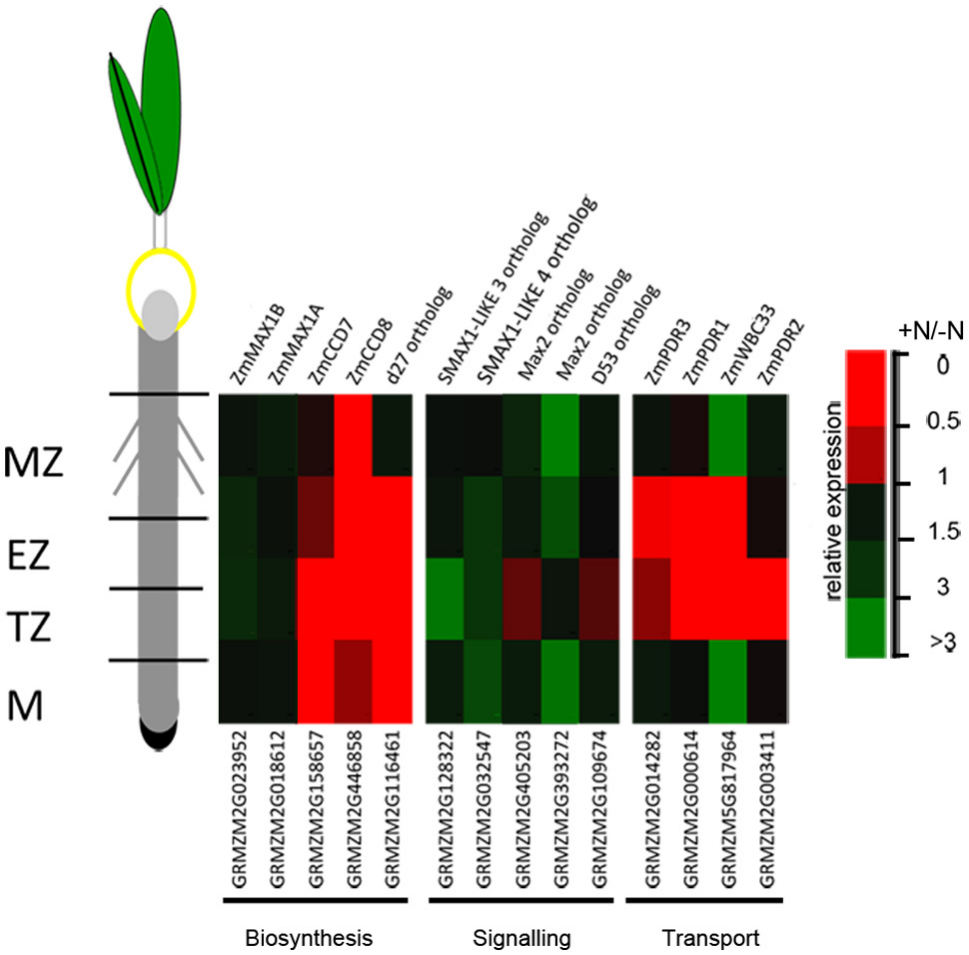
**Nitrate modulates SL-related gene expression.** Expression patterns of strigolactone biosynthesis, signaling and transport genes in maize root zones (M, meristem; TZ, transition zone; EZ, elongation zone; MZ, mature zone) expressed in response to the short term nitrate provision. Relative mRNA levels were normalized for individual genes relative to MEP (Manoli et al., 2012). Quantitative real-time PCR was performed in triplicate (three biological replicates) and mean values are shown as ratio (+NO_3_^-^/-NO_3_^-^).

The expression of a number of genes involved in SL biosynthesis (*ZmCCD7, ZmCCD8, ZmMAX1A, ZmMAX1B* and the rice ortholog d27) showed a significant down-regulation after 2 h of nitrate supply (Trevisan et al., 2015). Their transcript levels, together with that of other genes putatively implicated in SL signaling (*Arabidopsis MAX2, SMAX1-LIKE 3, SMAX1-LIKE 4* and rice *D53* orthologs respectively) and transport (*ZmPDR1, ZmPDR3, ZmWBC33* and *ZmPDR2*) were here measured in the four zones of the PR apices of seedlings grown for 24 h in a nitrate-depleted solution and then transferred for additional 2 h in a nitrate supplied or depleted solution. As observed for other genes (Trevisan et al., 2015), SL related genes also showed zone-specific responses. Genes involved in SL biosynthesis and transport showed all a clear inhibition of transcription in response to nitrate supply (**Figure 7**), with the maximum effect in cells of the TZ.

**FIGURE 8:**
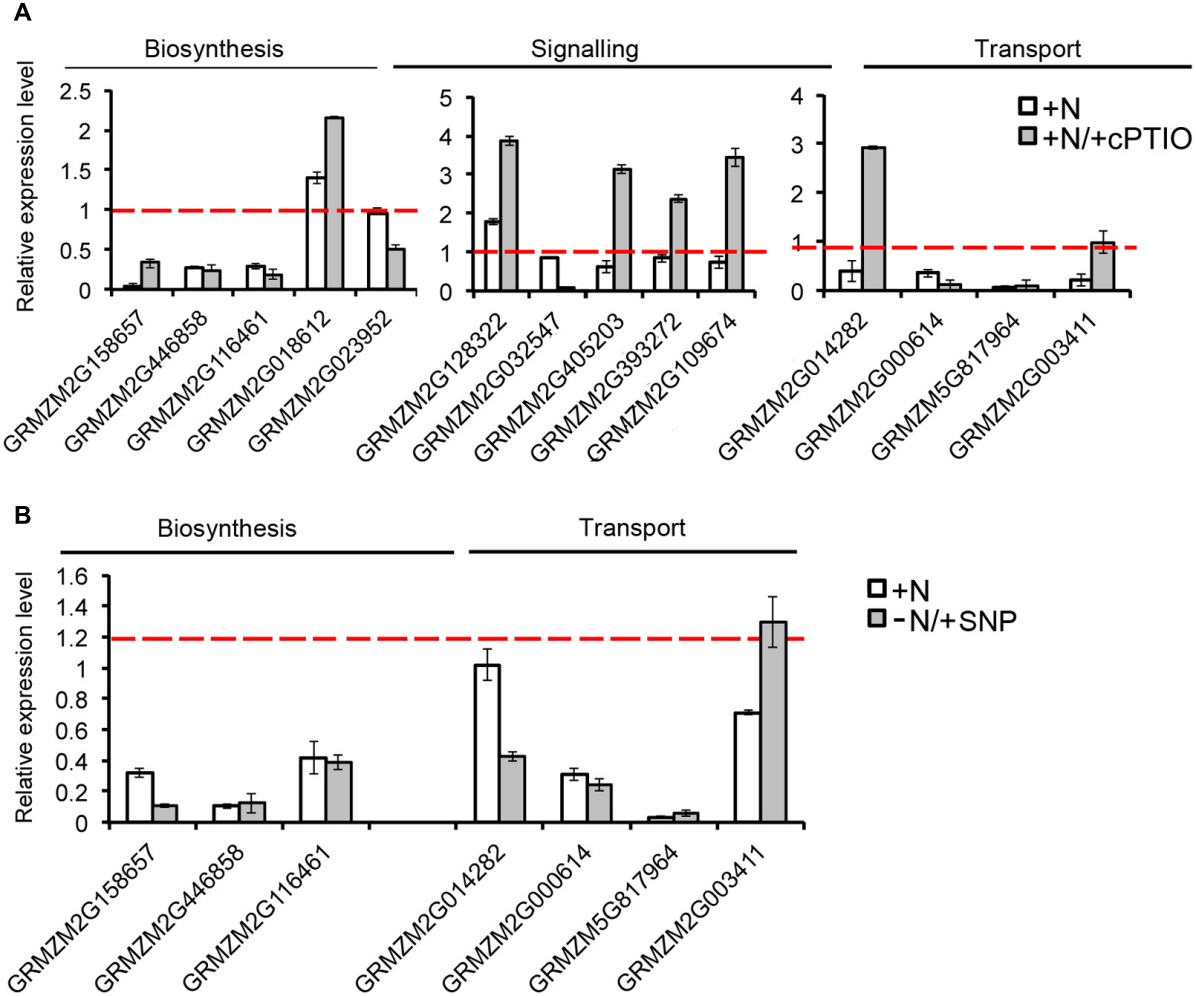
**Nitric oxide affects SL-related gene expression.** Expression patterns of strigolactone biosynthesis, signaling and transport genes in maize root TZ expressed in response to the short term nitrate provision (+N), and to nitrate in combination to cPTIO (+N/+cPTIO) **(A)**. Expression patterns of strigolactone biosynthesis and transport genes in maize root TZ expressed in response to the short term nitrate provision (+N), and to nitrate starvation in combination to SNP (-N/+SNP) **(B)**. For each gene, the expression level in the control plants (-N) was equal to 1 (red lines).

The strongest inhibitions exerted by 2 h of nitrate provision were detected on the transcription of *ZmCCD7* and *ZmCCD8* in the root TZ (0.2 and 0.03 fold changes, respectively). TZ was thus selected as target site to dissect the role of NO in the SL-mediated response. The addition of cPTIO to the nitrate supplied seedlings restrained or prevented the inhibition of expression produced by the presence of nitrate for *ZmCCD7*, *ZmPDR3*, *ZmWBC33* and for the *ZmPDR2*, ortholog of *PhPDR1* (**Figure 8A**). Accordingly, the application of a NO donor (SNP) to nitrate depleted seedlings clearly inhibited the transcription of a selection of SL-related genes (**Figure 8B**) which was similar to that observed for N-supplied roots. The recovery of genes transcription in presence of nitrate and cPTIO and its inhibition in presence of SNP in N-starved seedlings suggest a critical role of endogenous NO as a negative modulator of SL action.

### SL Actions are Involved in Early Regulation of Root Apex by Nitrate

**FIGURE 9:**
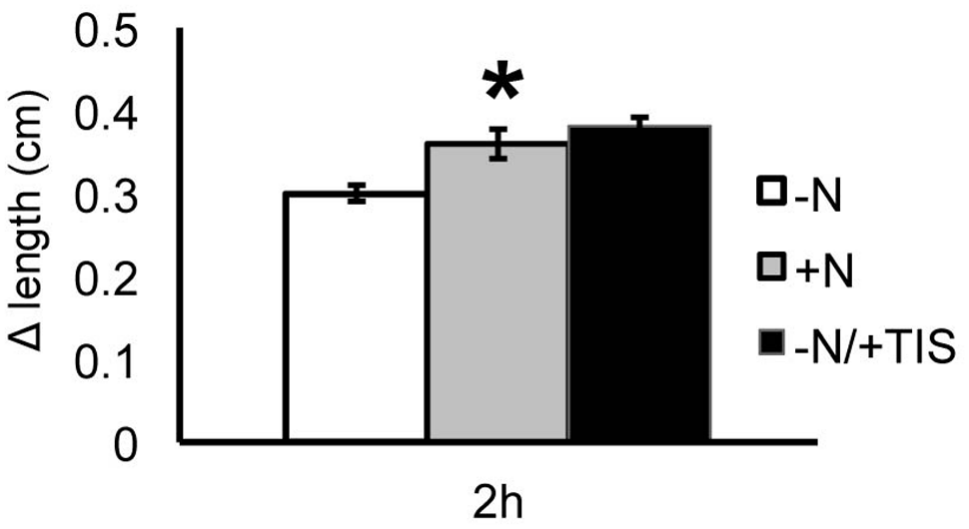
**Strigolactones and nitrate modulate *Zea mays* L. PR growth.** Two days maize seedlings were grown for 24 h in -NO_3_^-^ solution and then transferred to +NO_3_^-^ (gray bar), – NO_3_^-^ (white bar) or – NO_3_^-^ in combination to TIS108 (black bar). PR length was collected at T_0_ and after 2 h of nitrate depletion/provision. The time course of Δ length respect to the T_0_ was calculated. Data are means ± SE of three biological replicates. Asterisk indicates significant differences between the treatments, *P* < 0.01, based on ANOVA.

To deepen our understanding of the SL involvement in nitrate regulation of root response, the growth of PR was monitored also in the presence of an inhibitor of SL biosynthesis (TIS108). Seedlings were grown in a nitrate-depleted solution for 24 h and then transferred to a nitrate-deprived solution (negative control), to a nitrate-supplied one (positive control) and to a nitrate-free solution supplied with TIS108 (**Figure 9**).

When the SL inhibitor was provided for 2 h to nitrate-deprived seedlings the PR showed a growth increase similar to that observed after 2 h of nitrate provision and significantly higher than that measured for nitrate-depleted roots. This result suggests that the early induction of PR growth by nitrate is at least in part mediated by a down-regulation of SL production.

## Discussion

Roots represent the interface between plants and the surrounding soil environment and their plasticity is crucial for plants adaptation to a continuously changing environment. They are essential for mining water and nutrients from soil, thus strongly contributing to outline water and nutrients use efficiencies. For this reason, the study of roots provides critical knowledge to develop tools for sustainable agriculture.

The root apex functions as a dynamic sensory organ (Darwin, 1880) perceiving external stimuli, which are then processed into a root growth and developmental responses to environment (Sivaguru and Horst, 1998; Sivaguru et al., 1999; Kollmeier et al., 2000; Illés et al., 2006; Baluška et al., 2010; Mugnai et al., 2012; Baluška and Mancuso, 2013). The ability to re-orientate growth in response to environmental stimuli mainly derives from a unique feature of cells of the TZ which releases cells into the elongation region plastically, providing the growing root apices with an effective mechanisms to re-orientate root growth direction in response to environmental stimuli (Baluška et al., 2010).

Recently, Manoli et al. (2014) suggested that the NO production peaks at the TZ of nitrate-deprived roots immediately after the *de novo* nitrate supply stimulate root apex elongation, even though the downstream events triggering the root to elongate have still to be identified. Cytoskeletal proteins seem to represent a highly probable molecular target for NO signal and increasing evidences place NO among the key elements in the control of several cytoskeleton-mediated processes in plants (Kasprowicz et al., 2009; Wang et al., 2009; Yao et al., 2012). The effect of nitrate on PR growth seems to be more complex than expected and appears to be strongly variable depending on the NO_3_^-^ concentration, on the exposure times and on the species (Linkohr et al., 2002; Zhao et al., 2007; Gifford et al., 2008; Tian et al., 2008; Walch-Liu and Forde, 2008; Andrews et al., 2013).

In this paper, by monitoring the growth of PRs during the first 48 h of nitrate provision (**Figure 1A**), a dual effect is reported. Nitrate supplied to N-starved roots significantly stimulates growth during the first 2 h of treatment, as already indicated by Manoli et al. (2014), with no relevant effects after 6 h. However, an inhibitory effect was observed thereafter (24 and 48 h). These results support the hypothesis that growing root apices are able to differentially respond to nitrate depending on its availability in time and space.

To better understand the biological nature of events involved in the early boost of apex growth triggered by nitrate supply, the time-point corresponding to 2 h of nitrate supply was chosen and further experiments were conducted. The extents of cell division and cell expansion affect overall architecture of the root system (Benfey et al., 1993; Scheres et al., 2002; Jiang et al., 2006). Confocal analyses showed a lower number of cortical cells in TZ of roots supplied with nitrate for 2 h, but a clear increase of the cell size was also recorded, thus leading us to hypothesize that nitrate could stimulate the apex to grow by triggering cellular expansion, more than by accelerating cell division. The enzyme xyloglucan endotransglycosylase (XET) seems to play a predominant role in leading root apex growth, being involved in cell wall loosening during cell expansion (Pritchard et al., 1993; Vissenberg et al., 2000). XGs are hemicellulosic polysaccharides located in primary cell walls and firmly associated with cellulose microfibrils through hydrogen bonds to maintain the cell wall architecture (Fry, 1989; Hayashi, 1989; Sonobe et al., 2000). They have been recently demonstrated to affect both the stability of the microtubule cytoskeleton and the production and patterning of cellulose in primary cell walls, altering cell growth (Xiao et al., 2015). In our data, XET gene expression was significantly induced in the TZ upon short-time NO_3_^-^ provision and also XG immunofluorescence signal was stronger in the sample subjected to nitrate treatment, when compared to the negative control, suggesting a higher rate of XGs synthesis in response to the anion in maize root TZ (**Figures 2** and **3**).

In contrast to cellulose, which is synthesized on the plasma membrane, XGs are synthesized in the Golgi apparatus (Moore and Staehelin, 1988) and they can be actively internalized in root apex cells since, after BFA treatment, almost all XGs were removed from cell walls into BFA-compartments, revealing a high rate of XGs recycling in the root apex cells (Baluška et al., 2005). According to these authors in fact, internalization of cell wall macromolecules such as cross-linked pectins and XGs can be related to tight control of the mechanical properties of cell walls in the TZ, trough loosening wall structure thus enabling extensive elongation after ceasing mitotic divisions. Endocytosis of pectin-XG complexes and subsequent recycling would fulfill this requirement without loss of molecules and expending energy. In this context, endocytic vesicles filled with ready-to-use cell wall macromolecules would be ideally suited to provide “building blocks” for rapid formation of cell walls in cells that have ceased mitotic division and start to elongate. Data obtained in this work by supplying BFA to nitrate treated roots evidence the presence of a clear signal into BFA-induced compartments, even if a clear immunofluorescence was still visible also at cross walls (**Figure 2**). According to BFA action (reviewed by Nebenführ et al., 2002; Geldner et al., 2003; Šamaj et al., 2004), these results point out the hypothesis that nitrate might regulate root elongation, by modulating cytoskeleton-mediated cell wall deposition and recycling specifically in the TZ cells. Furthermore, the XG immunofluorescence was inhibited when cPTIO was supplied together with nitrate, suggesting a fascinating scenario in which nitrate might promote rapid cell elongation of root apex by regulating the synthesis and/or the turn-over of XGs within root transition cells, through the fine-tuning production of NO.

As far as auxin immunolabeling was concerned (**Figure 5**), in nitrate supplied roots IAA signal was strongly localized at the cross wall (end-poles) of TZ cells. In contrast, no such cross wall labeling was detected in the nitrate depleted roots, thus suggesting that IAA end-poles labeling was probably due to increased IAA fluxes triggered specifically by nitrate. Since it has been proposed that in the TZ auxin is transported via endocytosis of IAA molecules embedded within cell wall material like pectins and XGs (Baluška et al., 2005; Schlicht et al., 2006), the actin-dependent endocytosis could be implicated in regulating nitrate effects on root elongation, by targeting both XGs and auxin to plasma membrane. The identification of a number of transcripts involved in cytoskeletal organization (Trevisan et al., 2015; **Table 1**) and regulated by early nitrate supply, supports this suggestion.

Moreover, the polar localization of PINs also depends on vesicle trafficking between plasma membrane and endosomes which is dependent on F-actin (Geldner et al., 2001; Nagawa et al., 2012) and further studies indicate that PIN1 is co-transported with IAA via vesicular system (Šamaj et al., 2004; Schlicht et al., 2006). In NO_3_^-^-treated roots, PIN1 co-localizes with auxin at the cross walls (**Figure 5**). Interestingly in this respect, NO is known to inhibit *Arabidopsis* PR growth via reducing the acroptetal (rootward) transport of auxin mediated by PIN1, PIN3, and PIN7 (Fernández-Marcos et al., 2011; Liu et al., 2015). Our findings suggest that nitrate is able to increase IAA-fluxes involving at least in part PIN1 re-localization, likely by affecting endocytosis and vesicular trafficking. Consistent with this hypothesis, Tian et al. (2008) found that PR length showed a positive correlation with IAA content in maize roots. Moreover, XGs turnover is correlated with auxin-induced elongation and the gene expression of XETs and XET-related proteins (XTP) are also regulated by auxin (Vissenberg et al., 2000 and references therein). In this scenario, it is not surprising that the IAA and PIN-related genes expression is not significantly altered in response to nitrate, as showed in our data (**Figure 4**).

Among transcripts putatively involved in cytoskeletal organization (Trevisan et al., 2015) (**Table 1**) an unknown protein with an ArfGAP-like domain, with predicted orthology with the *Arabidopsis* ARF GAP-like zinc finger-containing protein AGD14 gene, has been chosen because its putative function is unprecedented in the nitrate network. Adenosine diphosphate Ribosylation Factors (ARFs) are members of the ARF family of the GTP-binding proteins and the ARF-GTPase activating proteins (GAPs) are a family of regulator proteins that induce hydrolysis of GTP-bound ARF, thus switching off the ARF cycle (Randazzo and Hirsch, 2004). ARF act as regulators of vesicular trafficking and actin remodeling (Du and Chong, 2011), but also interfere with auxin transport. The ectopic expression of *OsAGAP* in *Arabidopsis* alters the localization of AUX1 (Auxin Influx Carrier 1), which in turn controls auxin-dependent root growth in plants by regulating the vesicle trafficking and the cytoskeleton reorganization (Zhuang et al., 2006; Du and Chong, 2011). The transcription of ArfGAP-like of maize in meristem and TZ is strongly induced by nitrate (Trevisan et al., 2015) and mRNAs, here localized by ISH, are mainly expressed in epidermal cells, but also in pericycle and endodermis (**Figure 6**). These results allow us to hypothesize a contribution of this protein to the signaling pathway involving cytoskeleton modifications and auxin polar localization in controlling root adaptation to nitrate fluctuations.

Furthermore, the analysis of the transcriptional responses displayed by cells of the TZ in response to 2 h of nitrate provision (Trevisan et al., 2015) highlights also a putative role for SLs in triggering the root apex responses to nitrate. In the present work, the expression of a number of genes involved in SL biosynthesis, signaling and transport was measured in all the four zones of PR; revealing a significant inhibition of transcription for genes involved in the biosynthesis and transport after nitrate supply. This was particularly evident in the TZ, but not exclusively localized to this portion (see **Figure 7**).

Strigolactones have been initially identified as seed germination stimulants for parasitic plants (Cook et al., 1966) and lately described as branching factors for symbiotic arbuscular mycorrhizal (Akiyama et al., 2005). Nowadays, SLs are universally recognized as a novel class of plant hormones with numerous functions in plant development (Alder et al., 2012; Guan et al., 2012). They seem also to be involved in plant responses to several environment cues and nutrient availability. In particular phosphorus (P) and nitrogen (N) deficiency promote SLs production and exudation (Yoneyama et al., 2007a,b, 2013; López-Ráez and Bouwmeester, 2008; Umehara et al., 2010; Jamil et al., 2012; Ito et al., 2015). Considering their prominent role in regulating RSA (Kapulnik and Koltai, 2014), they seem to represent optimal candidates for signaling the nutrient status and transduce it in proper developmental responses, playing thus a crucial role in root adaptation to nutrient fluctuations.

Moreover, in roots, SLs increase cell number in the PR meristem (Ruyter-Spira et al., 2011; Koren et al., 2013), which is coherent with the higher number of cells scored in the TZ of maize nitrate starved roots. SLs exert their action on auxin transport through regulating PINs localization at the plasma membrane (Crawford et al., 2010; Koltai, 2015). In roots, the SL dependent PIN2 localization to plasma membrane was accompanied by an increase of PIN2 endocytosis and endosome trafficking in epidermal cells, and changes in actin-filament architecture and dynamics (Pandya-Kumar et al., 2014).

Based on our results, the auxin re-localization observed after nitrate supply in the TZ cells of maize root apex could, therefore, depend on the down-regulation of the SL biosynthesis occurring early in responses to nitrate provision. Moreover, the observation that by inhibiting SL biosynthesis for 2 h, the nitrate-depleted root presented a phenotype identical to that exhibited by 2 h nitrate fed seedlings, supports this hypothesis (**Figure 9**).

cPTIO scavenges NO and it restored, at least partially, the induction of expression of genes involved in biosynthesis and transport of SL in root apices of nitrate supplied seedlings (**Figure 8**), allowing us to hypothesize an involvement of NO upstream of SL in this pathway. This hypothesis is also supported by the down-regulation of the SL-related gene expression exerted by the NO donor SNP on nitrate deprived seedlings. A recent paper (Bharti and Bhatla, 2015) provided evidence of the role of endogenous NO as a negative modulator of CCD activity and therefore of SL biosynthesis during LR development in sunflower, giving more strength to our results. To conclude, our results highlight a complex effect of nitrate on the maize root apex development, with an earlier stimulating action and a later inhibiting one.

**FIGURE 10:**
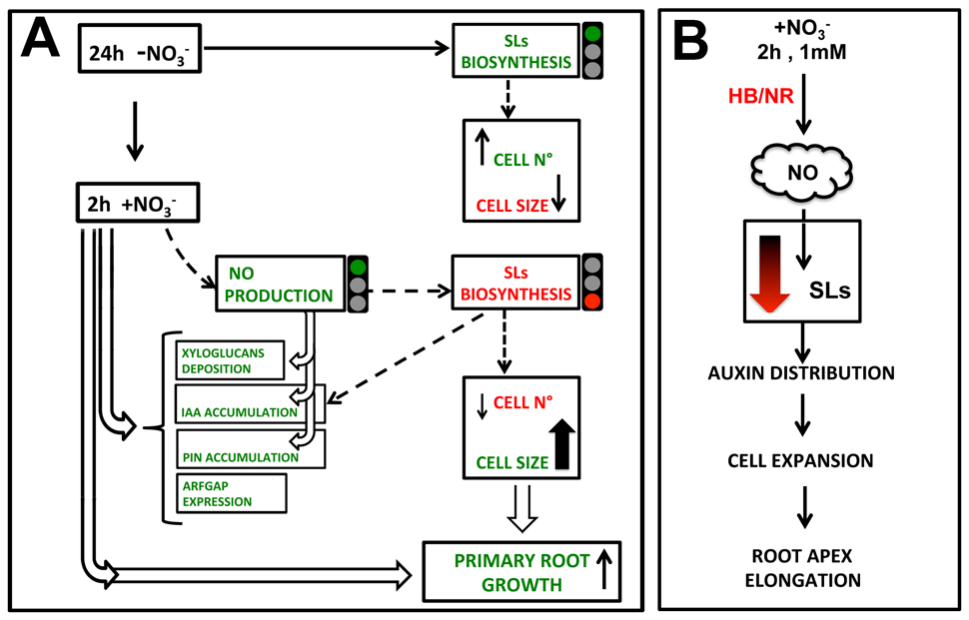
**Hypothetical model of how nitrate signaling might control PR growth, through triggering TZ enlargement.** 2 h nitrate provision would produce a NO boost which inhibits SL biosynthesis, leading to an auxin (IAA) and PIN1 re-distribution and to a reduction of cell division in favor of cell growth, also affecting xyloglucans deposition and cytoskeleton.

**Figure 10** shows an hypothetical scenario of how NO, auxin and SLs may cooperate in regulating the early response of maize root apex to nitrate. The NO burst, occurring specifically in the TZ of root apex immediately after the nitrate supply (Manoli et al., 2014) seems to operate by temporarily lowering, or turning off, the biosynthesis and transport of SL; thus indirectly affecting auxin polar transport and localization. This succession of events would trigger cell expansion and the early root apex growth observed after nitrate supply. Further and more specific evidences will be needed to better outline and complete this preliminary and partial picture.

## Author Contributions

AM and ST performed the experiments and wrote the manuscript. SQ conceived and designed the project, analyzed data, wrote the manuscript and obtained funds to support the project. BV and KY helped in performing immunolocalization experiments and in manuscript writing. FB contributed to concept the idea, helped in conducting various experiments and wrote the manuscript.

## Conflict of Interest Statement

The authors declare that the research was conducted in the absence of any commercial or financial relationships that could be construed as a potential conflict of interest.

## Supplementary Material

FIGURE S1 | Quantification of cortical cell number and calculation of the average cortex cell length in meristem of maize primary root.

## References

[B1] Akiyama K. Matsuzaki K. Hayashi H. (2005). Plant sesquiterpenes induce hyphal branching in arbuscular mycorrhizal fungi. *Nature* 435 824–827. 10.1038/nature03608 15944706

[B2] Alder A. Jamil M. Marzorati M. Bruno M. Vermathen M. Bigler P. (2012). The path from β-carotene to carlactone, a strigolactone-like plant hormone. *Science* 335 1348–1351. 10.1126/science.1218094 22422982

[B3] Andrews M. Raven J. A. Lea P. J. (2013). Do plants need nitrate? The mechanisms by which nitrogen form affects plants. *Ann. Appl. Biol.* 163 174–199. 10.1111/aab.12045

[B4] Baligar V. C. Fageria N. K. He Z. L. (2001). Nutrient use efficiency in plants. *Commun. Soil Sci. Plant Anal.* 32 921–950. 10.1081/CSS-100104098

[B5] Baluška F. Barlow P. W. Volkmann D. (1996). Complete disintegration of the microtubular cytoskeleton precedes its auxin-mediated reconstruction in postmitotic maize root cells. *Plant Cell Physiol.* 37 1013–1021. 10.1093/oxfordjournals.pcp.a029032 11536780

[B6] Baluška F. Kubica S. Hauskrecht M. (1990). Postmitotic ‘isodiametric’ cell growth in the maize root apex. *Planta* 181 269–274. 10.1007/BF00195876 24196802

[B7] Baluška F. Liners F. Hlavacka A. Schlicht M. Van Cutsem P. McCurdy D. W. (2005). Cell wall pectins and xyloglucans are internalized into dividing root cells and accumulate within cell plates during cytokinesis. *Protoplasma* 225 141–155. 10.1007/s00709-005-0095-5 16228896

[B8] Baluška F. Mancuso S. (2013). Root apex transition zone as oscillatory zone. *Front. Plant Sci.* 4:354 10.3389/fpls.2013.00354 PMC3788588 24106493

[B9] Baluška F. Mancuso S. Volkmann D. Barlow P. W. (2010). Root apex transition zone: a signalling-response nexus in the root. *Trends Plant Sci.* 15 402–408. 10.1016/j.tplants.2010.04.007 20621671

[B10] Benfey P. N. Linstead P. J. Roberts K. Schiefelbein J. W. Hauser M.-T. Aeschbacher R. A. (1993). Root development in *Arabidopsis*: four mutants with dramatically altered root morphogenesis. *Development* 119 57–70. 8275864 10.1242/dev.119.Supplement.57

[B11] Bharti N. Bhatla S. C. (2015). Nitric oxide mediates strigolactone signaling in auxin and ethylene-sensitive lateral root formation in sunflower seedlings. *Plant Signal. Behav.* 10:e1054087 10.1080/15592324.2015.1054087 PMC4622609 26076049

[B12] Blilou I. Xu J. Wildwater M. Willemsen V. Paponov I. Friml J. (2005). The PIN auxin efflux facilitator network controls growth and patterning in *Arabidopsis* roots. *Nature* 433 39–44. 10.1038/nature03184 15635403

[B13] Bouguyon E. Gojon A. Nacry P. (2012). Nitrate sensing and signaling in plants. *Sem. Cell. Dev. Biol.* 23 648–654. 10.1016/j.semcdb.2012.01.004 22273693

[B14] Cook C. E. Whichard L. P. Turner B. Wall M. E. Egley G. H. (1966). Germination of witchweed (*Striga lutea* Lour.): isolation and properties of a potent stimulant. *Science* 154 1189–1190. 10.1126/science.154.3753.1189 17780042

[B15] Correa-Aragunde N. Graziano M. Lamattina L. (2004). Nitric oxide plays a central role in determining lateral root development in tomato. *Planta* 218 900–905. 10.1007/s00425-003-1172-7 14716561

[B16] Crawford S. Shinohara N. Sieberer T. Williamson L. George G. Hepworth J. (2010). Strigolactones enhance competition between shoot branches by dampening auxin transport. *Development* 137 2905–2913. 10.1242/dev.051987 20667910

[B17] Darwin C. R. (1880). *The Power of Movements in Plants.* London: John Murray Available at: http://darwin-online.org.uk/

[B18] Du C. Chong K. (2011). ARF-GTPase activating protein mediates auxin influx carrier AUX1 early endosome trafficking to regulate auxin dependent plant development. *Plant Signal. Behav.* 6 1644–1646. 10.4161/psb.6.11.17755 22057332 PMC3329325

[B19] Erisman J. W. Galloway J. N. Seitzinger S. Bleeker A. Dise N. B. Petrescu A. M. R. (2013). Consequences of human modification of the global nitrogen cycle. *Philos. Trans. R. Soc. B.* 368:20130116 10.1098/rstb.2013.011 PMC3682738 23713116

[B20] Erisman J. W. Sutton M. A. Galloway J. Klimont Z. Winiwarter W. (2008). How a century of ammonia synthesis changed the world. *Nat. Geosci.* 1 636–639. 10.1038/ngeo325

[B21] Fernández-Marcos M. Sanz L. Lewis D. R. Muday G. K. Lorenzo O. (2011). Nitric oxide causes root apical meristem defects and growth inhibition while reducing PIN-FORMED 1 (PIN1)-dependent acropetal auxin transport. *Proc. Natl. Acad. Sci. U.S.A.* 108 18506–18511. 10.1073/pnas.1108644108 22021439 PMC3215072

[B22] Forde B. G. (2002). Local and long-range signaling pathways regulating plant responses to nitrate. *Annu. Rev. Plant Biol.* 53 203–224. 10.1146/annurev.arplant.53.100301.135256 12221973

[B23] Forestan C. Farinati S. Varotto S. (2012). The maize PIN gene family of auxin transporters. *Front. Plant Sci.* 3:16 10.3389/fpls.2012.00016 PMC3355596 22639639

[B24] Forestan C. Varotto S. (2010). PIN1 auxin efflux carriers localization studies in *Zea mays*. *Plant Signal. Behav.* 5 436–439. 10.4161/psb.5.4.11339 20383059 PMC7080414

[B25] Fowler D. Coyle M. Skiba U. Sutton M. A. Cape J. N. Reis S. (2013). The global nitrogen cycle in the twenty-first century. *Philos. Trans. R. Soc. B.* 368:20130164 10.1098/rstb.2013.0164 PMC3682748 23713126

[B26] Fry S. (1989). The structure and functions of xyloglucan. *J. Exp. Bot.* 40 1–11. 10.1093/jxb/40.1.1

[B27] Galloway J. N. Leach A. M. Bleeker A. Erisman J. W. (2013). A chronology of human understanding of the nitrogen cycle. *Philos. Trans. R. Soc. B.* 368:20130120 10.1098/rstb.2013.0120 PMC3682740 23713118

[B28] Geldner N. Anders N. Wolters H. Keicher J. Kornberger W. Muller P. (2003). The *Arabidopsis* GNOM ARF-GEF mediates endosomal recycling, auxin transport, and auxin-dependent plant growth. *Cell* 112 219–230. 10.1016/S0092-8674(03)00003-5 12553910

[B29] Geldner N. Friml J. Stierhof Y. D. Jürgens G. Palme K. (2001). Auxin transport inhibitors block PIN1 cycling and vesicle trafficking. *Nature* 413 425–428. 10.1038/35096571 11574889

[B30] Gifford M. L. Dean A. Gutiérrez R. A. Coruzzi G. M. Birnbaum K. D. (2008). Cell-specific nitrogen responses mediate developmental plasticity. *Proc. Natl. Acad. Sci. U.S.A.* 105 803–808. 10.1073/pnas.0709559105 18180456 PMC2206617

[B31] Guan J. C. Koch K. E. Suzuki M. Wu S. Latshaw S. Petruff T. (2012). Diverse roles of strigolactone signaling in maize architecture and the uncoupling of a branching-specific subnetwork. *Plant Physiol.* 160 1303–1317. 10.1104/pp.112.204503 22961131 PMC3490586

[B32] Hayashi T. (1989). Xyloglucans in the primary cell wall. *Annu. Rev. Plant Physiol. Plant Mol. Biol.* 40 139–168. 10.1146/annurev.pp.40.060189.001035

[B33] Hirel B. Le Gouis J. Ney B. Gallais A. (2007). The challenge of improving nitrogen use efficiency in crop plants: towards a more central role for genetic variability and quantitative genetics within integrated approaches. *J. Exp. Bot.* 58 2369–2387. 10.1093/jxb/erm097 17556767

[B34] Ho C. H. Lin S. H. Hu H. C. Tsay Y. F. (2009). CHL1 functions as a nitrate sensor in plants. *Cell* 138 1184–1194. 10.1016/j.cell.2009.07.004 19766570

[B35] Illés P. Schlicht M. Pavlovkin J. Lichtscheidl I. Baluška F. Ovečka M. (2006). Aluminium toxicity in plants: internalization of aluminium into cells of the transition zone in *Arabidopsis* root apices related to changes in plasma membrane potential, endosomal behaviour, and nitric oxide production. *J. Exp. Bot.* 57 4201–4213. 10.1093/jxb/erl197 17085753

[B36] Ishikawa H. Evans M. L. (1992). Induction of curvature in maize roots by calcium or by thigmostimulation: role of the postmitotic isodiametric growth zone. *Plant Physiol.* 100 762–768. 10.1104/pp.100.2.762 11537870 PMC1075624

[B37] Ito S. Nozoye T. Sasaki E. Imai M. Shiwa Y. Shibata-Hatta M. (2015). Strigolactone regulates anthocyanin accumulation, acid phosphatases production and plant growth under low phosphate condition in *Arabidopsis*. *PLoS ONE* 10:e0119724 10.1371/journal.pone.0119724 PMC4368578 25793732

[B38] Ito S. Umehara M. Hanada A. Kitahata N. Hayase H. Yamaguchi S. (2011). Effects of triazole derivatives on strigolactone levels and growth retardation in rice. *PLoS ONE* 6:e21723 10.1371/journal.pone.0021723 PMC3132747 21760901

[B39] Jamil M. Charnikhova T. Houshyani B. van Ast A. Bouwmeester H. J. (2012). Genetic variation in strigolactone production and tillering in rice and its effect on Striga hermonthica infection. *Planta* 235 473–484. 10.1007/s00425-011-1520-y 21947621 PMC3288373

[B40] Jiang K. Ballinger T. Li D. Zhang S. Feldman L. (2006). Role for mitochondria in the establishment and maintenance of the maize root quiescent center. *Plant Physiol.* 140 1118–1125. 10.1104/pp.105.071977 16443698 PMC1400572

[B41] Kapulnik Y. Koltai H. (2014). Strigolactone involvement in root development, response to abiotic stress, and interactions with the biotic soil environment. *Plant Physiol.* 166 560–569. 10.1104/pp.114.244939 25037210 PMC4213088

[B42] Kasprowicz A. Szuba A. Volkmann D. Baluška F. Wojtaszek P. (2009). Nitric oxide modulates dynamic actin cytoskeleton and vesicle trafficking in a cell type-specific manner in root apices. *J. Exp. Bot.* 60 1605–1617. 10.1093/jxb/erp033 19261922 PMC2671617

[B43] Kollmeier M. Felle H. H. Horst W. J. (2000). Genotypical differences in aluminum resistance of maize are expressed in the distal part of the transition zone. Is reduced basipetal auxin flow involved in inhibition of root elongation by aluminum? *Plant Physiol.* 122 945–956. 10.1104/pp.122.3.945 10712559 PMC58931

[B44] Koltai H. (2015). Cellular events of strigolactone signalling and their crosstalk with auxin in roots. *J. Exp. Bot.* 66 4855–4861. 10.1093/jxb/erv178 25900617

[B45] Koren D. Resnick N. Gati E. Belausov E. Weininger S. Kapulnik Y. (2013). Strigolactone signaling in the endodermis is sufficient to restore root responses and involves SHORT HYPOCOTYL 2 (SHY2) activity. *New Phytol.* 198 866–874. 10.1111/nph.12189 23425316

[B46] Krouk G. Crawford N. M. Coruzzi G. M. Tsay Y. F. (2010). Nitrate signaling: adaptation to fluctuating environments. *Curr. Opin. Plant Biol.* 13 265–272. 10.1016/j.pbi.2009.12.003 20093067

[B47] Linkohr B. I. Williamson L. C. Fitter A. H. Leyser H. M. (2002). Nitrate and phosphate availability and distribution have different effects on root system architecture of *Arabidopsis*. *Plant J.* 29 751–760. 10.1046/j.1365-313X.2002.01251.x 12148533

[B48] Liu W. Li R. J. Han T. T. Cai W. Fu Z. W. Lu Y. T. (2015). Salt stress reduces root meristem size by nitric oxide-mediated modulation of auxin accumulation and signaling in *Arabidopsis*. *Plant Physiol.* 168 343–356. 10.1104/pp.15.00030 25818700 PMC4424022

[B49] Livak K. J. Schmittgen T. D. (2001). Analysis of relative gene expression data using real-time quantitative PCR and the 2(–Delta Delta C(T)) method. *Methods* 25 402–408. 10.1006/meth.2001.1262 11846609

[B50] López-Arredondo D. L. Leyva-González M. A. Alatorre-Cobos F. Herrera-Estrella L. (2013). Biotechnology of nutrient uptake and assimilation in plants. *Int. J. Dev. Biol.* 57 595–610. 10.1387/ijdb.130268lh 24166442

[B51] López-Bucio J. Cruz-Ramírez A. Herrera-Estrella L. (2003). The role of nutrient availability in regulating root architecture. *Curr. Opin. Plant Biol.* 6 280–287. 10.1016/S1369-5266(03)00035-9 12753979

[B52] López-Ráez J. A. Bouwmeester H. (2008). Fine-tuning regulation of strigolactone biosynthesis under phosphate starvation. *Plant Signal. Behav.* 3 963–965. 10.4161/psb.6126 19704420 PMC2633743

[B53] Manoli A. Begheldo M. Genre A. Lanfranco L. Trevisan S. Quaggiotti S. (2014). NO homeostasis is a key regulator of early nitrate perception and root elongation in maize. *J. Exp. Bot.* 65 185–200. 10.1093/jxb/ert358 24220653 PMC3883287

[B54] Manoli A. Sturaro A. Trevisan S. Quaggiotti S. Nonis A. (2012). Evaluation of candidate reference genes for qPCR in maize. *J. Plant Physiol.* 169 807–815. 10.1016/j.jplph.2012.01.019 22459324

[B55] Marciano D. P. R. O. Toledo-Ramos F. Neiva-Alvim M. Magalhaes J. R. Costa França M. G. (2010). Nitric oxide reduces the stress effects of aluminum on the process of germination and early root growth of rice. *J. Plant Nutr. Soil Sci.* 173 885–891. 10.1002/jpln.200900312

[B56] Masi E. Ciszak M. Comparini D. Monetti E. Pandolfi C. Azzarello E. (2015). The electrical network of maize root apex is gravity dependent. *Sci. Rep.* 5 7730 10.1038/srep07730 PMC4295110 25588706

[B57] Méndez-Bravo A. Raya-González J. Herrera-Estrella L. López-Bucio J. (2010). Nitric oxide is involved in alkamide-induced lateral root development in *Arabidopsis*. *Plant Cell Physiol.* 51 1612–1626. 10.1093/pcp/pcq117 20685967

[B58] Miller A. J. Fan X. Orsel M. Smith S. J. Wells D. M. (2007). Nitrate transport and signalling. *J. Exp. Bot.* 58 2297–2306. 10.1093/jxb/erm066 17519352

[B59] Moore P. J. Staehelin L. A. (1988). Immunogold localization of the cell-wall-matrix polysaccharides rhamnogalacturonan I and xyloglucan during cell expansion and cytokinesis in *Trifolium pratense* L.; implication for secretory pathways. *Planta* 174 433–445. 10.1007/BF00634471 24221558

[B60] Mugnai S. Azzarello E. Baluška F. Mancuso S. (2012). Local root apex hypoxia induces NO-mediated hypoxic acclimation of the entire root. *Plant Cell Physiol.* 53 912–920. 10.1093/pcp/pcs034 22422934

[B61] Mugnai S. Pandolfi C. Masi E. Azzarello E. Monetti E. Comparini D. (2014). Oxidative stress and NO signalling in the root apex as an early response to changes in gravity conditions. *Biomed. Res. Int.* 2014:834134 10.1155/2014/834134 PMC4150467 25197662

[B62] Nagawa S. Xu T. Lin D. Dhonukshe P. Zhang X. Friml J. (2012). ROP GTPase-dependent actin microfilaments promote PIN1 polarization by localized inhibition of clathrin-dependent endocytosis. *PLoS Biol.* 10:e1001299 10.1371/journal.pbio.1001299 PMC3317906 22509133

[B63] Nebenführ A. Ritzenthaler C. Robinson D. G. (2002). Brefeldin A: deciphering an enigmatic inhibitor of secretion. *Plant Physiol.* 130 1102–1108. 10.1104/pp.011569 12427977 PMC1540261

[B64] Nonis A. Ruperti B. Pierasco A. Canaguier A. Adam-Blondon A. F. Di Gaspero G. (2008). Neutral invertases in grapevine and comparative analysis with *Arabidopsis*, poplar and rice. *Planta* 229 129–142. 10.1007/s00425-008-0815-0 18800225

[B65] Nonis A. Scortegagna M. Nonis A. Ruperti B. (2011). PRaTo: a web-tool to select optimal primer pairs for qPCR. *Biochem. Biophys. Res. Commun.* 415 707–708. 10.1016/j.bbrc.2011.10.148 22086170

[B66] Pandya-Kumar N. Shema R. Kumar M. Mayzlish-Gati E. Levy D. Zemach H. (2014). Strigolactone analog GR24 triggers changes in PIN2 polarity, vesicle trafficking and actin filament architecture. *New Phytol.* 202 1184–1196. 10.1111/nph.12744 24571327

[B67] Prinsi B. Negri A. S. Pesaresi P. Cocucci M. Espen L. (2009). Evaluation of protein pattern changes in roots and leaves of *Zea mays* plants in response to nitrate availability by two-dimensional gel electrophoresis analysis. *BMC Plant Biol.* 9:113 10.1186/1471-2229-9-113 PMC2744680 19698183

[B68] Pritchard J. Hetherington P. R. Fry S. C. Tomos A. D. (1993). Xyloglucan endotransglycosylase activity, microfibril orientation and the profiles of cell wall properties along growing regions of maize roots. *J. Exp. Bot.* 44 1281–1289. 10.1093/jxb/44.8.1281

[B69] Randazzo P. A. Hirsch D. S. (2004). Arf GAPs: multifunctional proteins that regulate membrane traffic and actin remodelling. *Cell. Signal.* 16 401–413. 10.1016/j.cellsig.2003.09.012 14709330

[B70] Raun W. R. Johnson G. V. (1999). Improving nitrogen use efficiency for cereal production. *Agron. J.* 91 357–363. 10.2134/agronj1999.00021962009100030001x

[B71] Robertson G. P. Vitousek P. M. (2009). Nitrogen in agriculture: balancing the cost of an essential resource. *Annu. Rev. Environ. Resour.* 34 97–125. 10.1146/annurev.environ.032108.105046

[B72] Rozen S. Skaletsky H. (2000). Primer3 on the WWW for general users and for biologist programmers. *Methods Mol. Biol.* 132 365–386. 10.1385/1-59259-192-2:365 10547847

[B73] Ruiz Herrera L. F. Shane M. W. López-Bucio J. (2015). Nutritional regulation of root development. *Wiley Interdiscip Rev. Dev. Biol.* 4 431–443. 10.1002/wdev.183 25760021

[B74] Ruyter-Spira C. Kohlen W. Charnikhova T. van Zeijl A. van Bezouwen L. de Ruijter N. (2011). Physiological effects of the synthetic strigolactone analog GR24 on root system architecture in *Arabidopsis*: another belowground role for strigolactones? *Plant Physiol.* 155 721–734. 10.1104/pp.110.166645 21119044 PMC3032462

[B75] Šamaj J. Baluška F. Voigt B. Schlicht M. Volkmann D. Menzel D. (2004). Endocytosis, actin cytoskeleton, and signaling. *Plant Physiol.* 135 1150–1161. 10.1104/pp.104.040683 15266049 PMC519036

[B76] Sanz L. Albertos P. Mateos I. Sánchez-Vicente I. Lechón T. Fernández-Marcos M. (2015). Nitric oxide (NO) and phytohormones crosstalk during early plant development. *J. Exp. Bot.* 66 2857–2868. 10.1093/jxb/erv213 25954048

[B77] Sanz L. Fernández-Marcos M. Modrego A. Lewis D. R. Muday G. K. Pollmann S. (2014). Nitric oxide plays a role in stem cell niche homeostasis through its interaction with auxin. *Plant Physiol.* 166 1972–1984. 10.1104/pp.114.247445 25315603 PMC4256006

[B78] Scheres B. Benfey P. Dolan L. (2002). “Root development,” in *The Arabidopsis Book*, eds Somerville C. R. Meyerowitz E. M. (Rockville, MD: American Society of Plant Biologists). 10.1199/tab.0101 PMC3243376 22303222

[B79] Schlicht M. Strnad M. Scanlon M. J. Mancuso S. Hochholdinger F. Palme K. (2006). Auxin immunolocalization implicates vesicular neurotransmitter-like mode of polar auxin transport in root apices. *Plant Signal. Behav.* 1 122–133. 10.4161/psb.1.3.2759 19521492 PMC2635008

[B80] Sivaguru M. Baluška F. Volkmann D. Felle H. H. Horst W. J. (1999). Impacts of aluminum on the cytoskeleton of the maize root apex. short-term effects on the distal part of the transition zone. *Plant Physiol.* 119 1073–1082. 10.1104/pp.119.3.1073 10069846 PMC32089

[B81] Sivaguru M. Horst W. J. (1998). The distal part of the transition zone is the most aluminum-sensitive apical root zone of maize. *Plant Physiol.* 116 155–163. 10.1104/pp.116.1.155

[B82] Sivaguru M. Liu J. Kochian L. V. (2013). Targeted expression of SbMATE in the root distal transition zone is responsible for sorghum aluminum resistance. *Plant J.* 76 297–307. 10.1111/tpj.12290 23865685

[B83] Sonobe S. Nakayama N. Shimmen T. Sone Y. (2000). Intercellular distribution of subcellular organelles revealed by antibody against xyloglucan during cell cycle in tobacco BY-2 cells. *Protoplasma* 213 218–227. 10.1007/BF01282159

[B84] Tian Q. Y. Chen F. J. Liu J. Zhang F. S. Mi G. H. (2008). Inhibition of maize root growth by high nitrate supply is correlated with reduced IAA levels in roots. *J. Plant Physiol.* 165 942–951. 10.1016/j.jplph.2007.02.011 17928098

[B85] Trevisan S. Manoli A. Begheldo M. Nonis A. Enna M. Vaccaro S. (2011). Transcriptome analysis reveals coordinated spatiotemporal regulation of hemoglobin and nitrate reductase in response to nitrate in maize roots. *New Phytol.* 192 338–352. 10.1111/j.1469-8137.2011.03822.x 21762167

[B86] Trevisan S. Manoli A. Quaggiotti S. (2014). NO signaling is a key component of the root growth response to nitrate in *Zea mays* L. *Plant Signal. Behav.* 9:e28290 10.4161/psb.28290 PMC4091522 24613869

[B87] Trevisan S. Manoli A. Ravazzolo L. Botton A. Pivato M. Masi A. (2015). Nitrate sensing by the maize root apex transition zone: a merged transcriptomic and proteomic survey. *J. Exp. Bot.* 66 3699–3715. 10.1093/jxb/erv165 25911739 PMC4473975

[B88] Umehara M. Hanada A. Magome H. Takeda-Kamiya N. Yamaguchi S. (2010). Contribution of strigolactones to the inhibition of tiller bud outgrowth under phosphate deficiency in rice. *Plant Cell Physiol.* 51 1118–1126. 10.1093/pcp/pcq084 20542891 PMC2900824

[B89] Vidal E. A. Gutiérrez R. A. (2008). A systems view of nitrogen nutrient and metabolite responses in *Arabidopsis*. *Curr. Opin. Plant Biol.* 5 521–529. 10.1016/j.pbi.2008.07.003 18775665

[B90] Vidal E. A. Moyano T. C. Riveras E. Contreras-López O. Gutiérrez R. A. (2013). Systems approaches map regulatory networks downstream of the auxin receptor AFB3 in the nitrate response of *Arabidopsis thaliana* roots. *Proc. Natl. Acad. Sci. U.S.A.* 110 12840–12845. 10.1073/pnas.1310937110 23847199 PMC3732920

[B91] Vissenberg K. Martinez-Vilchez I. M. Verbelen J. P. Miller J. G. Fry S. C. (2000). In vivo colocalization of xyloglucan endotransglycosylase activity and its donor substrate in the elongation zone of *Arabidopsis* roots. *Plant Cell* 12 1229–1237. 10.1105/tpc.12.7.1229 10899986 PMC149061

[B92] Walch-Liu P. Forde B. G. (2008). Nitrate signalling mediated by the NRT1.1 nitrate transporter antagonises L-glutamate-induced changes in root architecture. *Plant J.* 54 820–828. 10.1111/j.1365-313X.2008.03443.x 18266918

[B93] Walch-Liu P. Ivanov I. I. Filleur S. Gan Y. Remans T. Forde B. G. (2006). Nitrogen regulation of root branching. *Ann. Bot.* 97 875–881. 10.1093/aob/mcj601 16339770 PMC2803407

[B94] Wang Y. Chen T. Zhang C. Hao H. Lui P. Zheng M. (2009). Nitric oxide modulates the influx of extracellular Ca^2+^ and actin filament organization during cell wall construction in *Pinus bungeana* pollen tubes. *New Phytol.* 182 851–862. 10.1111/j.1469-8137.2009.02820.x 19646068

[B95] Wang Y. Y. Hsu P. K. Tsay Y. F. (2012). Uptake, allocation and signaling of nitrate. *Trends Plant Sci.* 17 458–467. 10.1016/j.tplants.2012.04.006 22658680

[B96] Xiao C. Zhang T. Zheng Y. Cosgrove D. J. Anderson C. T. (2015). Xyloglucan deficiency disrupts microtubule stability and cellulose biosynthesis in *Arabidopsis*, altering cell growth and morphogenesis. *Plant Physiol.* 170 234–249. 10.1104/pp.15.01395 26527657 PMC4704587

[B97] Yang Z. B. Geng X. He C. Zhang F. Wang R. Horst W. J. (2014). TAA1-regulated local auxin biosynthesis in the root-apex transition zone mediates the aluminum-induced inhibition of root growth in *Arabidopsis.* *Plant Cell* 26 2889–2904. 10.1105/tpc.114.127993 25052716 PMC4145121

[B98] Yao L.-L. Pei B.-L. Zhou Q. Li Y.-Z. (2012). NO serves as a signaling intermediate downstream of H_2_O_2_ to modulate dynamic microtubule cytoskeleton during responses to VD-toxins in *Arabidopsis*. *Plant Signal. Behav.* 7 174–177. 10.4161/psb.18768 22353875 PMC3405694

[B99] Yoneyama K. Kisugi T. Xie X. Yoneyama K. (2013). “Chemistry of strigolactones: why and how do plants produce so many strigolactones?,” in *The Molecular Microbial Ecology of the Rhizosphere*, ed. de Bruijn F. J. (Hoboken, NJ: John Wiley & Sons, Inc.).

[B100] Yoneyama K. Xie X. Kusumoto D. Sekimoto H. Sugimoto Y. Takeuchi Y. (2007a). Nitrogen deficiency as well as phosphorus deficiency in sorghum promotes the production and exudation of 5-deoxystrigol, the host recognition signal for arbuscular mycorrhizal fungi and root parasites. *Planta* 227 125–132. 10.1007/s00425-007-0600-5 17684758

[B101] Yoneyama K. Yoneyama K. Takeuchi Y. Sekimoto H. (2007b). Phosphorus deficiency in red clover promotes exudation of orobanchol, the signal for mycorrhizal symbionts and germination stimulant for root parasites. *Planta* 225 1031–1038. 10.1007/s00425-006-0410-1 17260144

[B102] Yu M. Lamattina L. Spoel S. H. Loake G. J. (2014). Nitric oxide function in plant biology: a redox cue in deconvolution. *New Phytol.* 202 1142–1156. 10.1111/nph.12739 24611485

[B103] Zhao D. Y. Tian Q. Y. Li L. H. Zhang W. H. (2007). Nitric oxide is involved in nitrate-induced inhibition of root elongation in *Zea mays*. *Ann. Bot.* 100 497–503. 10.1093/aob/mcm142 17709366 PMC2533613

[B104] Zhuang X. Jiang J. Li J. Ma Q. Xu Y. Xue Y. (2006). Over-expression of OsAGAP, an ARF-GAP, interferes with auxin influx, vesicle trafficking and root development. *Plant J.* 48 581–591. 10.1111/j.1365-313X.2006.02898.x 17059407

