# Supplementary material for: Nitric Oxide-Mediated Maize Root Apex Responses to Nitrate are Regulated by Auxin and Strigolactones
Source: Front Plant Sci. 2016 Jan 22;6:1269. doi: 10.3389/fpls.2015.01269 (PMC4722128; doi:10.3389/fpls.2015.01269)
Supplement: Supplementary file 1 [file Image_1.PDF]

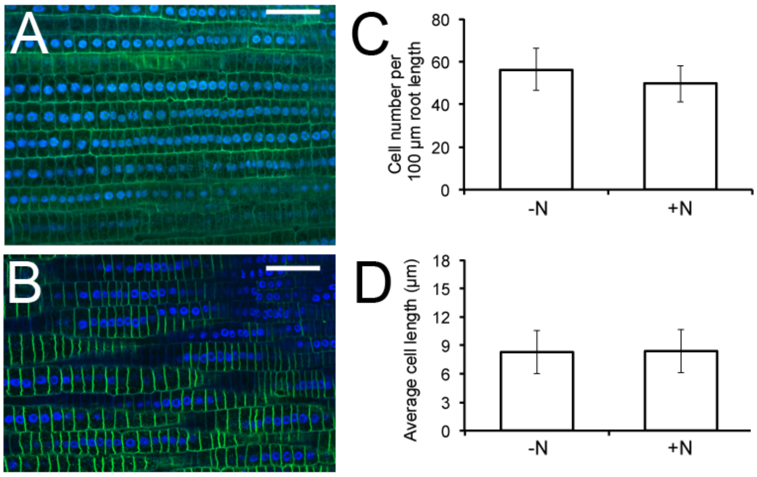

Supplementary Figure S1
Nitrate availability did not modulates *Zea mays* L. meristem growth.
Two days maize seedlings were grown for 24 h in -NO_3^- solution and then transferred to + NO_3^- (A) or – NO_3^- (B) medium.
Confocal images were analyzed to determine the cortical cell number in the meristem zone of each plant after 2h of NO_3^- supply (A) depletion (B). Histograms summarizing quantification of cortical cell number (C) and calculation of the average cortex cell length (D) in the cortex of maize root meristem. Results are presented as mean±SE from three experiments (n=5–10). Bar=100 µm. Asterisk indicates significant differences, $P < 0.01$, based on ANOVA.
