# Supplementary material for: Nitric Oxide-Mediated Maize Root Apex Responses to Nitrate are Regulated by Auxin and Strigolactones
Source: Front Plant Sci. 2016 Jan 22;6:1269. doi: 10.3389/fpls.2015.01269 (PMC4722128; doi:10.3389/fpls.2015.01269)
Supplement: Supplementary file 2 [file Table_1.DOCX]

**Supplementary Table S1**

Primers used in qRT-PCR

| **Gene** | **Annotation** | **Forward (5’-3’)** | **Reverse (5’-3’)** |
| --- | --- | --- | --- |
| ZmXET1 | GRMZM2G026980 | GCATGGATGTCGCAGGAG | TTGCTCTCTGTATCCGATCAA |
| ZmCCD7 | GRMZM2G158657 | CGGCACAACACAAACAGAAA | GCGACCGGAATAGGAGGT |
| ZmCCD8 | GRMZM2G446858 | AGAAAGGTGTCTCTGCTGCT | CTATGGGCTCGCTCACATGA |
| D27 ortholog | GRMZM2G116461 | GCGACGAAACAGAGCTACTC | TTCTTGAAGGTTCCGGGACA |
| SMAX1-LIKE 3 ortholog | GRMZM2G128322 | AGTCATTGAAGGAACCGTATGC | TACACCGGCGATCCAATTCT |
| SMAX1-LIKE 4 ortholog | GRMZM2G032547 | AAAATGGCGGGAAAGGGAAG |  |
| GTPase-activating protein | GRMZM2G145008 | CGTCTTGGACTTGGCATGTT | CCCGGTAGTGCTGAATAATCA |
| ZmMAX1B | GRMZM2G023952 | TCGGTCCCAGGATTTGCATC | AGCACAACACCGAACTGAAA |
| ZmMAX1A | GRMZM2G018612 | AGAAGGGCGTCAAACTCGTA | CACTGACTGCAAGCAATGGA |
| MAX2 ortholog | GRMZM2G405203 | GAACAAGACCGGCATCCAAC | TTAACTCGTCAGGCCTCCAG |
| MAX2 ortholog | GRMZM2G393272 | CAATCTCCGCAAGCTCGTG | CTGCCGGCTCAAAAGGTTC |
| D53 ortholog | GRMZM2G109674 | ACCCTGAGACTGGTTTCCTG | CTACAGTACGGTGGGTGGTG |
| ZmPDR3 | GRMZM2G014282 | AGTTCTAGCAGCCTTCCCAA | ACCTTTTGTATCAGAGGGCAA |
| ZmPDR1 | GRMZM2G000614 | GGAAACCCGATCAGCAGGT | GCAGTAAAGCCAGCCAACAC |
| ZmPDR2 | GRMZM2G003411 | ATCAGGCTGGAAGATGACGG | GGATGACGCCCTCCCTTATC |
| ZmWBC33 | GRMZM5G817964 | GGGGCTCTACAAGAACGAGT | TTGACCGCCTCACCTACTTT |
